# Quantum Anharmonic Calculations of Vibrational Spectra for Water Adsorbed on Titania Anatase(101) Surface: Dissociative versus Molecular Adsorption

**DOI:** 10.1021/acs.jpcc.2c02137

**Published:** 2022-07-19

**Authors:** Marco Cazzaniga, Marco Micciarelli, Fabio Gabas, Fabio Finocchi, Michele Ceotto

**Affiliations:** † Dipartimento di Chimica, Universitá degli Studi di Milano, via Golgi 19, 20133 Milano, Italy; ‡ Sorbonne Université, CNRS, Institut des NanoSciences de Paris (INSP), 4 Place Jussieu, Paris F- 75005, France

## Abstract

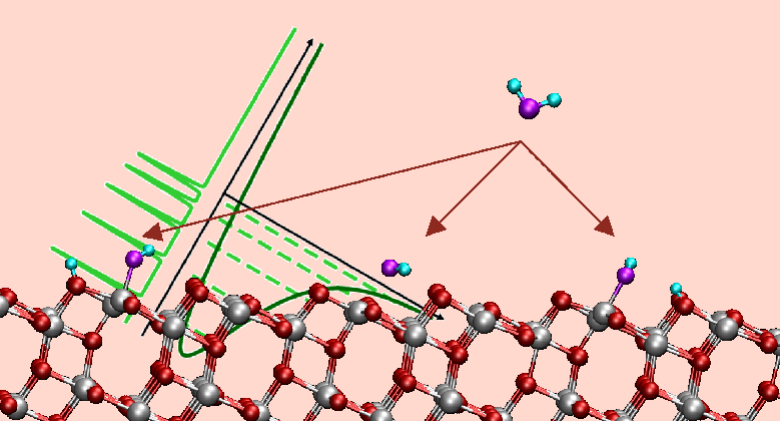

The interaction of water molecules and hydroxyl groups with titanium dioxide (TiO_2_) surfaces is ubiquitous and very important in anatase nanoparticle photocatalytic processes. Infrared spectroscopy, assisted by ab initio calculations of vibrational frequencies, can be a powerful tool to elucidate the mechanisms behind water adsorption. However, a straightforward comparison between measurements and calculations remains a challenging task because of the complexity of the physical phenomena occurring on nanoparticle surfaces. Consequently, severe computational approximations, such as harmonic vibrational ones, are usually employed. In the present work we partially address this complexity issue by overcoming some of the standard approximations used in theoretical simulations and employ the Divide and Conquer Semiclassical Initial Value Representation (DC-SCIVR) molecular dynamics. This method allows to perform simulations of vibrational spectra of large dimensional systems accounting not only for anharmonicities, but also for nuclear quantum effects. We apply this computational method to water and deuterated water adsorbed on the ideal TiO_2_ anatase(101) surface, contemplating both the molecular and the dissociated adsorption processes. The results highlight not only the presence of an anharmonic shift of the frequencies in agreement with the experiments, but also complex quantum mechanical spectral signatures induced by the coupling of molecular vibrational modes with the surface ones, which are different in the hydrogenated case from the deuterated one. These couplings are further analyzed by exploiting the mode subdivision performed during the divide and conquer procedure.

## Introduction

Understanding photocatalytic processes is important for their numerous potential applications in many fields, such as environmental remediation (e.g., mineralization of pollutants),^1^ and energy conversion (e.g., facilitating the process of water splitting).^2,3^ Among the several materials, titanium dioxide (TiO_2_) is one of the most promising, thanks to its abundance, high stability, and nontoxicity. Even if the rutile polymorph is the thermodynamically more stable one, it is the anatase polymorph that is more interesting for photocatalytic applications. However, anatase is available mainly in the form of nanoparticles, where the size is nanoscopic and quite complicated. Given the nanostructures involved in the photocatalytic processes, an atomistic comprehension of the mechanisms of molecular adsorption on TiO_2_ surfaces, both from an experimental and theoretical point of view, becomes crucial.^4−11^

Water adsorption is of tantamount importance in photocatalysis because it is ubiquitous and unavoidable, given water vapor in the air. During the synthesis of TiO_2_ and other oxide nanoparticles, water is an omnipresent component, despite the several treatments that have been proposed to dehydrate the nanoparticles. It is then realistic to consider that some amount of water or hydroxyl groups are present on the nanoparticle surfaces. This motivated a wide effort to understand the process of water adsorption on oxide surfaces and nanoparticles.^7,12,13^

Among the variety of experimental approaches adopted to investigate the behavior of water and hydroxyl groups on titanium nanoparticles, infrared spectroscopy can provide access to the vibrational frequencies of the adsorbed species and possibly allow an understanding of how water vibrational frequencies are modified by the interaction with the nanoparticle or other coadsorbed molecules.^6,14^ Unfortunately, the complexity and the variety of processes at nanoparticle surfaces cause the experimental spectra to be rather difficult to interpret. Even if an adsorption peak around 1615–1640 cm^–1^ can be assigned to the bending mode of the adsorbed molecular water, the IR spectra presents a broad adsorption band with several secondary structures in the frequency region, which is typical of OH stretchings (2500–3800 cm^–1^) and that are difficult to interpret.^15−25^ Roughly speaking, the stretching modes of an adsorbed molecular water are generally attributed to the lower frequency part of this absorption band, while the hydroxyl stretching has been assumed to be responsible for the high energy part of the band. This is clearly a simplified picture since, for example, the hydroxyl stretching can have a lower frequency when ascribed either to the formation of hydrogen bonds or to surface oxygen coordination or even in a water monolayer adsorption structure. In addition, the complex morphology of the nanoparticles exhibits not only different surfaces (mainly the (101) and (001) in the case of anatase TiO_2_), but also complex morphologies originated, for example, from high index facets, edges or corners, and defects.^26^ Additionally, side residuals from the synthesis and experimental limitations in controlling the environment during the measurements can further complicate the spectra interpretation.

In this paper, we focus on the relevance of anharmonic and quantum contributions to the vibrational spectra of water adsorption on anatase(101) surfaces and, more generally, in the field of molecular adsorption. We try to reach this goal by simulating the vibrational frequencies of the system composed by a water molecule and titania. Theoretical calculations can help in providing not only an interpretation of spectroscopy measurements, but also new atomistic physical insights. A standard harmonic estimation of the vibrational frequencies, based on Density Functional Theory (DFT) Potential Energy Surfaces (PES), can be inaccurate enough to deal with such a complex system, where intermolecular gas–surface interactions are important.^27,28^ While, from one side, it is difficult to improve the accuracy of the description of the electron–electron interaction, as provided by standard DFT functionals for these large periodic systems, also overcoming the harmonic approximations can still be a challenge.^29^ The inclusion of anharmonic contributions can be achieved, for example, by a scaled harmonic approximation^23^ or by means of the estimation of the velocity or dipole moment autocorrelation function on top of a molecular dynamic trajectory.^30,31^ Quantum mechanical effects can also be included, relying on approximate approaches, as applied to TiO_2_ surfaces,^32^ or can be addressed with complex theoretical approaches, as tested in metallic surfaces.^33^ These approaches, when applied to water molecules adsorbed on the TiO_2_ surfaces, can suffer from inaccuracies originating from the arbitrary choices of the scaling factors or from the reduced dimensionality of the calculation, as well as from the model employed to describe the nanoparticle surfaces. Consequently, when interpreting a complex experimental spectra, as in this case, it can become difficult to distinguish if the mismatch between theory and measurement can be ascribed to a limitation of the adopted approximation at the level of theory or to the complex morphology of the nanoparticles, which can hardly be addressed in an exhaustive way.

For these reasons, we tackle this spectroscopic problem with a quantum vibrational mechanic method that we have recently introduced,^34^ which can be an alternative with respect to the quantum mechanical approaches presented by Manzhos and Ihara.^29^ The Divide and Conquer Semiclassical Initial Value Representation (DC-SCIVR) approach^34,35^ to the semiclassical molecular dynamics is a useful tool that relies on classical Born–Oppenheimer trajectories and is able to address this issue since it has the capability of describing quantum nuclear effects, like zero point energy motion, overtones, and combination bands, which are supposed to be important in describing the water molecule adsorption. The basic idea that permits the application of such a computationally expensive approach to a large dimensional system as a titania slab with adsorbed molecules is to split the full problem in a set of vibrational degrees of freedom subspaces. In addition, the application of this approach to the problem of adsorption can be further facilitated from the fact that one is generally interested only in the high energy modes originating from the adsorbed molecule and a few phonon ones. Consequently, the simulation can be limited to the subspace, including the adsorbate modes and a few interacting ones.^36^ Moreover, a side result of the DC-SCIVR, which can be achieved by an inspection of the modes coupled in the same subspace, is the possibility to discuss the mechanism of interaction between the adsorbed molecule and the surface motion.

Specifically, in this paper, we apply DC-SCIVR to calculate the power spectra of water adsorbed on the stoichiometric TiO_2_ Anatase (101) surface, which is the most stable among the anatase surfaces. We restrict our analysis on a single molecule adsorbed on the surface thus neglecting the effect of the coverage and of the interaction between several water molecules at the level of vibrational properties. In addition, we will consider for adsorption the most energetically favorable configuration, which corresponds to the molecule on the 5-fold coordinated titanium atoms (Ti_5c_). There is also the possibility that the adsorption process yields the dissociation of the adsorbed molecule, thus leaving a hydroxyl group on the Ti_5c_ site and the remaining hydrogen bonds with a two coordinate surface oxygen (O_2c_). We also contemplate these cases and we investigate the difference and similarities in the vibrational behavior of these different adsorption mechanisms in conjunction with the molecular hydrogen deuteration isotopic effect by performing similar DC-SCIVR calculations for the adsorption of deuterated water. A comparison of the results performed at different levels of theory (harmonic vs classical anharmonic vs semiclassical approximate quantum mechanical) will provide an estimate of the effect of some adopted approximations and, hopefully, help in unraveling some of the open questions in the interpretation of the experiments.

First, we briefly summarize the basics of the DC-SCIVR approach and the computational parameters used in this work and then we present our results. These address, in order, the adsorption geometry, the adsorbate vibrational frequencies, and the description of the vibrational couplings. A general discussion and comparison with the available measurements follows.

## Computational Methods

### Ab Initio Calculation Computational Details

All ab initio calculations are performed with the open source Quantum-Espresso (Q-E)^37,38^ suite of codes at the level of Density Functional Theory (DFT) with a Perdew–Burke–Ernzerhof (PBE) parametrization for the exchange and correlation functional. The PBE functional is known to provide a good description of hydrogen bonds, although the ionic and covalent bonds are usually slightly too long, which leads to underestimate the harmonic frequencies. In addition we adoptultrasoft pseudopotentials for the description of the core electrons. The plane wave energy cutoff was set to 40 Ry for the wave functions and 400 Ry for the density, and the Brillouin zone (BZ) was sampled using the Γ point only. This choice is justified by the size of the supercell, which is 7.58 Å along the [010] direction and 10.33 Å along [1̅01]. This limited k-point sampling does not affect the values of the harmonic frequencies (see Table S4). The surface has been modeled through a supercell generated by starting from the TiO_2_ anatase bulk ab initio equilibrium geometry and lattice constants. The supercell has been cut along the plane defined by the (101) Miller indexes. The choice of the (101) surface is motivated by the fact that it is the thermodynamic most stable one. Then, the adsorption geometries were determined by a further geometry optimization of the surface atoms and the adsorbants, by adopting a threshold of 10^–7^ Ry on the total energy and 10^–7^ Ry/*a*_0_ on the forces. The slab thickness consists of four Ti layers, with the two deepest ones frozen at the bulk geometry, while the other coordinates are free to relax to their equilibrium values. A separation between periodic replica of the slabs has been achieved by inserting ∼10 Å layer of vacuum. The most stable site for adsorption on the TiO_2_ anatase(101) surface is on top of the Ti_5c_ atoms, and our choice of supercell makes four Ti_5c_ sites available for adsorption. Thus, the adsorption of a single molecule per supercell corresponds to a coverage Θ = 0.25. The relative stability of the different adsorption geometries has been estimated though the evaluation of the Binding Energy (BE)
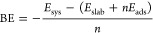
1where *E*_sys_ is the energy of the interacting surface–adsorbant system, *E*_slab_ is the energy of the bare slab, *E*_ads_ is the energy of the isolated adsorbed molecule in its equilibrium configuration, and *n* is the number of the adsorbed molecules (*n* = 1 in our case).

The vibrational frequencies have been estimated at different level of accuracy with the purpose of highlighting the anharmonic effects. As a reference, the harmonic estimation of vibrational frequencies have been determined through the Density-Functional Perturbation Theory (DFPT)^39^ implementation available in Q-E. In this way, we compute the equilibrium Hessian matrix and, after its diagonalization, the matrix for coordinate transformation from Cartesian to normal modes. The nonanalytic term which corrects the long wavevector limit, that is, *q* → 0 limit, of the dynamical matrix for polar crystals has been neglected in the evaluation of the Hessian matrix, since it presents a limited effect on the vibrational frequencies of the adsorbate, as we have verified. We also checked that the choice of limiting the BZ sampling to the Γ point, the choice of a limited slab thickness, and of neglecting the Hubbard *U* parameter in the Hamiltonian or Empirical Dispersions yield to a limited effect on the geometries and harmonic frequency estimates (see Tables S1–S5 in the Supporting Information). In order to decouple the motion of the relaxed atoms from that of the atoms that are frozen in their bulk positions, we set the Hessian matrix elements of the latter ones to zero.

### Classical Power Spectrum Calculation

We adopt a molecular dynamics approach to improve the accuracy with respect to the harmonic frequency estimates, including anharmonic effects. A classical estimation of anharmonic frequencies can be obtained from the Fourier transform of the velocity autocorrelation function. We run an ab initio molecular dynamics trajectory to calculate the velocity autocorrelation function. Specifically, we employ the time-averaging filtering technique^40^ to improve the numerical convergence with respect to the number of time-steps according to the following formula:
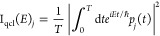
2where *p*_*j*_(*t*) is the *j*-esime component of the normal mode **p**(*t*) momentum vector. A similar formula can be employed in atomic Cartesian coordinates. This kind of simulation will reproduce the fundamental frequency of each mode, including the anharmonic contributions. The Born–Oppenheimer molecular dynamics (BOMD) has been performed with an in-house adaptation of Plane-Wave Self-Consistent Field (PWScf) module to integrate the equation of motion using the symplectic velocity-Verlet algorithm. The BOMD equation of motion have been solved in the NVE ensemble for 2500 iterations with a time-step of 10 au (0.242 fs), thus yielding 0.6 ps of dynamics. The kinetic energy is initialized at the harmonic zero-point energy (ZPE), and for this reason, the method is also called “quasi-classical”.

### Semiclassical Power Spectrum Calculation

Given a representation of the underlying PES, the semiclassical power spectrum reproduces all the vibrational eigenvalues on an absolute scale, including overtones and resonances. In contrast, the quasi-classical approximation is composed of a series of fundamental frequencies only. In the present context, the semiclassical approach can reveal the combination bands arising from the coupling between the modes of the adsorbate and the surface phonons.

Our quantum mechanical vibrational levels are determined though the Divide-and-Conquer Semiclassical Initial Value Representation (DC-SCIVR) method that we developed recently.^34,35,41−51^ Here we report only the working equations of the DC-SCIVR approach for the calculation of vibrational spectra. The reader can find further details in ref (52), and further details can be found in regard to the DC-SCIVR application to adsorption studies in ref (36). We present the method starting from the Multiple Coherent states Semiclassical Initial Value Representation (MC-SCIVR) version of the semiclassical molecular dynamics,^53−58^ on which the DC-SCIVR approach is based. The MC-SCIVR vibrational power spectra I(*E*) are calculated with the following sum over *N*_traj_ classical trajectories:
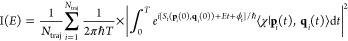
3where *T* is the total trajectory simulation time, *S*_*t*_(**p**(0),**q**(0)) is the classical action, ϕ_*t*_ is the phase of the Herman–Kluk pre-exponential factor *C*_*t*_(**p**(0), **q**(0))
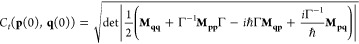
4where **M**_**qq**_ = ∂**q**(*t*)/∂**q**(0) and so on are the monodromy matrix blocks.

In eq 3, |**p**(*t*),**q**(*t*)⟩ is a coherent state of the following type:^59−64^
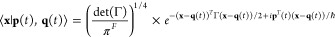
5In eqs 4 and 5, Γ is approximated to be a diagonal width matrix for bound state calculations, with coefficients usually equal to the square root of the harmonic vibrational frequencies, and the time evolution of *C*_*t*_(**p**(0),**q**(0)) is computed through the solution of the equation of motion for the monodromy matrix ***M***, for which the knowledge of the Hessian matrix along the MD trajectory is required. The advantage of the MC-SCIVR method with respect to other quantum methods is that a suitable choice of initial conditions for the MD trajectories and of the reference coherent states |χ⟩ can enhance the spectral features corresponding to molecular vibrational excitations and avoid the burden of the phase space integration required by other semiclassical molecular dynamics approaches. Specifically, the trajectory initial conditions should be set to provide the system an energy close to a given quantum vibrational eigenvalue, whose condition can be satisfied by relying on its harmonic estimate, that is, **p**_eq_^2^/2*m* = *ℏ***ω**(**n** + 1/2). Additionally, the corresponding excitation can be selected by a suitable choice of the reference superposition of coherent states of the type:
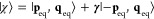
6where **γ** is a vector such that when all its elements are equal to 1, the ZPE peak and all even overtone intensities are enhanced,^56^ while when one of its component is equal to −1, the fundamental and the odd overtones of that mode component are enhanced. Unfortunately the MC-SCIVR is limited to a few tens of degrees of freedom calculations. For this reason, we have introduced the DC-SCIVR approach,^34,35,41^ whose strategy is to split the full dimensional *N*_vib_ problem into one at reduced dimensions (*M* < *N*_vib_). This partition is 2-fold because it allows for faster numerical convergence with respect to the full dimensional calculation and also for the identification of the different spectroscopic signals. Therefore, the power spectrum I(*E*) of eq 3 is obtained as the composition of partial spectra Ĩ(*E*), computed in an *M*-dimensional subspace of the full *N*_vib_-dimensional space. The DC-SCIVR power spectrum formula is
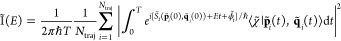
7where all the quantities with the tilde superscript are evaluated by projecting the full dimensional ones onto the M-subspace. In this way, we can drastically reduce the dimensionality of the semiclassical calculation. For most of the quantities in eq 7, the projection onto each subspace starting from the full dimensional one consists of taking the related vector components, except for the classical action *S̃*_*t*_, due to the nonseparability of the potential energy term. We obviate to this issue by assuming the following expression for the reduced dimensional potential
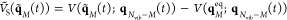
8where  is the equilibrium values of the positions for the degrees of freedom that do not belong to the M-dimensional subspace S. The subspace subdivision should pair the stronger coupled modes in the same subspace. Among the different approaches developed in our group,^65,66^ in the present work we will employ the “Hessian” method^52,65^ for the vibrational space subdivision. Specifically, the magnitude of the coupling between different modes is estimated by calculating the off-diagonal elements of the average Hessian *H̅*_*ij*_ matrix (in absolute value and normal mode coordinates) over some representative trajectory steps, that is, . Then, the subspace subdivision is obtained by fixing a threshold value ϵ under which the *H̅*_*ij*_ is set to zero. In this way the averaged Hessian can be divided into sub-blocks such that all normal modes within the same matrix sub-block are also in the same subspace. The Hessian subblocks of interest are calculated at each time-step.

The DC-SCIVR implementation for the case of the adsorbed molecules on solid surfaces is described in ref (36). Here, we adopt the same computational approach. Specifically, we calculate the averaged Hessian over 20 full-dimensional Hessian matrices computed within DFPT at time-steps uniformly distributed along the trajectory. We think that this number of Hessian matrices are representative enough of the trajectory geometries, as already shown elsewhere.^36^ To identify the preferable choice for each subspace dimension, we performed a DC-SCIVR power spectrum calculation of increasing subspace dimensionality until the quality of the spectrum was satisfactory. The quality has been evaluated by considering mainly that there should not be any signal at energy values lower than the ZPE value and that the position and the intensity of each peak remains unaffected by increasing the subspace dimensionality. Once we have identified how the full dimensional vibrational subspace is divided into subspaces, we compute the ingredients in eq 7 that are necessary for the determination of DC-SCIVR spectra. These are the projected potential, as in eq 8, and the Hessian at each time-step. To reduce the computational cost, we calculate only the Hessian elements of the subblocks that correspond to the vibrational subspace of the adsorbate modes and the mostly coupled phonons. Most of the phonon mode is weakly coupled with the adsorbant modes, and we are not interested in their spectroscopic signal. For the Hessian evaluation, we adopt a finite difference approach starting from the forces obtained from the ab initio code and an atomic displacement  with η_0_ equal to 5 × 10^–4^ and  being the harmonic frequency of the mode.

## Results and Discussion

### Geometry Considerations

**Figure 1:**
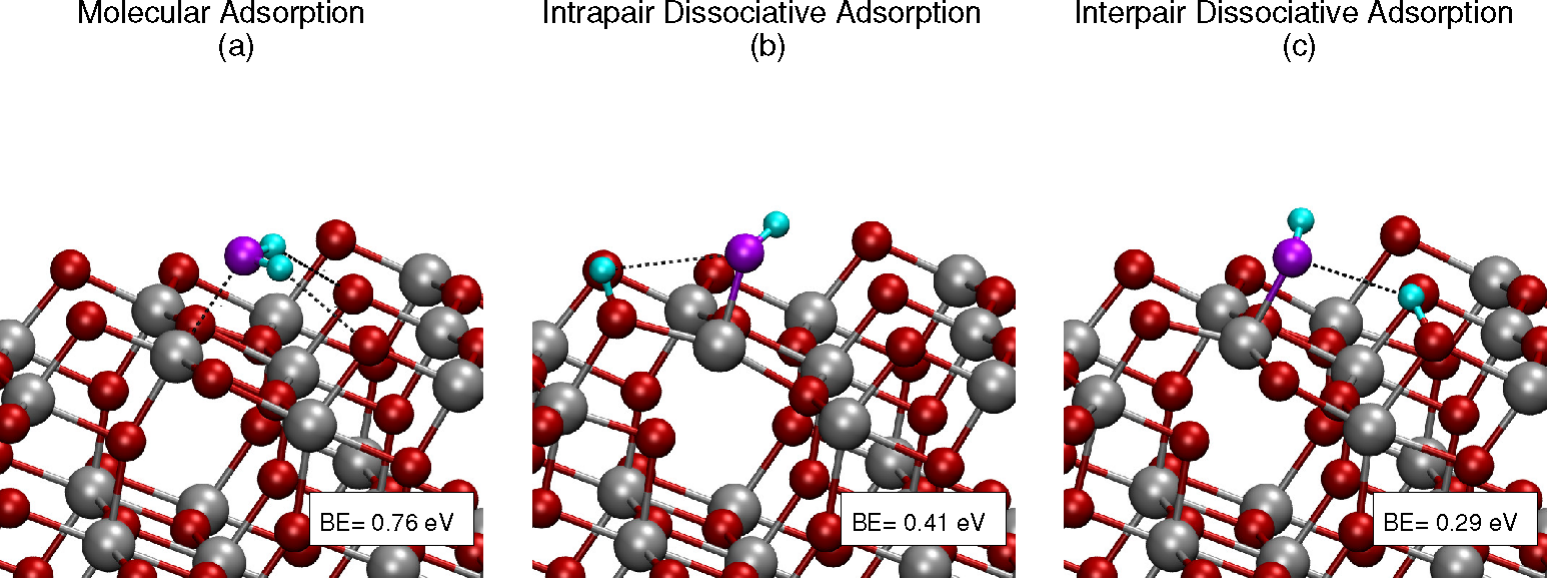
Optimized geometries of molecular and dissociative H_2_O adsorption on the TiO_2_ anatase(101) surface. Titanium atoms are in gray, surface oxygen in red, molecular oxygen in violet, and hydrogen in cyan. Binding energies are reported in the boxes.

We start by determining which are the most stable water adsorption sites on the (101) anatase TiO_2_ surface. These sites correspond to the Ti_5c_ atoms. Our geometry optimization includes the adsorbant and part of the slab, and it yields the conformations reported in Figure 1 (or in Figure S1 in the Supporting Information where the slab periodicity is more evident).We have identified three main different adsorption geometries, in agreement with the literature.^67−75^ Two geometries involve the molecule dissociation where a hydrogen atom is bonded to a surface O_2c_ site, while the remaining one is the molecular adsorption. In this last case, the water oxygen O_w_ atom is bonded to a Ti_5c_ site at a distance with ∼2.3 Å bond length and the two hydrogen atoms point toward two surface O_2c_ sites forming hydrogen bonds. The geometry parameters for the molecular adsorption are reported in Table 1. The comparison with the available literature values shows a quantitative agreement.^27,71−73^

**Table 1:** Relevant Geometry Parameters and Binding Energies (BE) for Molecular H_2_O Adsorption on the TiO_2_ Anatase(101) Surface

	layers	DFT *V*_*xc*_	θ	*r*(Ti_5c_–O_w_) (Å)	*r*(O_w_–H) (Å)	d(O_2c_–H) (Å)	α(H–O_w_–H) (°)	BE^a^ (eV)
Q-E present	4L	PBE	0.25	2.289	0.984	2.284	103.89	0.76
CASTEP^68^	8L	PBE		2.236	0.995	2.101	102.585	0.948
CASTEP^67^	4L	PBE		2.245	0.993	2.122		0.916
Q-E^70^		PBE	0.17	2.30		2.26		0.71
Q-E^69^		PBE	0.17	2.300		2.257/2.263		0.720
Q-E^71^	4L	PBE	0.17			2.30		0.730
Crystal14^72^	4L	PBE	0.25			2.25		0.71
Crystal14^72^	4L	HSE06	0.25			2.29		0.84
Q-E^73^		PBE		2.32		2.35		0.73
Q-E^76^		PBE		2.31		2.34		0.67
CPMD^74^		PW91		2.28	1.02	1.88/1.96		0.74
Crystal17^75^	8L	PBE0	0.25			2.28/2.43		0.78

a Experimental value 0.5–0.7 eV.^77^

Table 2 reports the geometry parameters for the dissociative adsorption. We distinguish two configurations, depending on the mutual positions of the hydroxyl groups that arise from the molecule dissociation: in the “intrapair” configuration (Figure 1b), O_2c_–H and O_w_–H bind the same Ti_5c_. In the “interpair” configuration, reported in Figure 1, they bind different titanium atoms. The relevant interatomic distances and the binding energies are detailed in Table 2. In the interpair conformation a weak hydrogen bond is formed between the hydrogen adsorbed on the surface O_2c_ site and the dissociated hydroxyl group (d(O_w_–H_diss_) = 2.487 Å), while in the intrapair configuration, the hydrogen bond is absent or very weak (d(O_w_–H_diss_) = 2.680 Å).

**Table 2:** Relevant Geometrical Parameters and Binding Energies (BE) for Dissociative H_2_O Adsorption on the TiO_2_ Anatase(101) Surface

	layers	DFT *V*_*xc*_	θ	*r*(Ti_5c_–O_w_) (Å)	*r*(O_w_–H) (Å)	d(O_2c_–H) (Å)	d(O_w_–H_diss_) (Å)	α(O_w_–H_diss_–O_2c_) (°)	BE (eV)
Intrapair Dissociative Adsorption									
Q-E present	4L	PBE	0.25	1.824	0.976	0.973	2.680	79.578	0.41
CASTEP^68^	8L	PBE		1.816	0.983	1.039			0.658
CASTEP^67^	4L	PBE		1.819	0.981	0.978	2.549		0.622
Crystal14^72^	4L	PBE	0.25				2.71		0.38
Crystal14^72^	4L	HSE06	0.25				2.65		0.54
CPMD^74^		PW91		1.83	0.99				0.30
Crystal17^75^	8L	PBE0	0.25				2.58		0.47
Interpair Dissociative Adsorption									
Q-E present	4L	PBE	0.25	1.854	0.975	0.982	2.487	138.282	0.29
CASTEP^67^	4L	PBE		1.835	0.977	0.985	2.598		0.5
Crystal14^72^	4L	PBE	0.25				2.75		0.34
Crystal14^72^	4L	HSE06	0.25				2.83		0.49
Q-E^76^		PBE		1.83			2.66		0.32
CPMD^74^		PW91		1.85	1.00	1.00	2.39		0.23
Crystal17^75^	8L	PBE0	0.25				2.74		0.42

By comparing the value of the BE of the three type of adsorptions, we find that the molecular adsorption is the most stable one, followed by the intrapair dissociative configuration, which is slightly more stable than the interpair one. The larger binding energy of the molecular adsorption configuration is consistent with the formation of two quite strong hydrogen bonds between H_w_ and two surface O_2c_, which is not the case upon water dissociation.

### Harmonic Frequencies Calculations

The calculations of harmonic vibrational frequencies is the first step toward the understanding of the relevance of the anharmonic contributions to the vibrational spectra. As a reference, the harmonic frequencies are reported in Table 3. When comparing with the literature ones, which are also calculated only at harmonic level, we find some differences, as well as there are significant differences between the literature values. We think that these discrepancies at the harmonic level of theory are mainly due to the difference computational setup, such as the slab dimension. However, for each type of calculation, we observe for the molecular adsorption the normal mode displacements of the adsorbate to be of the type of symmetric/asymmetric stretches and bending, as in the gas phase. While the bending frequency remains almost unchanged upon adsorption, the two stretching frequencies are smaller both in the hydrogenated and deuterated case. This red-shift can be ascribed to the formation of the hydrogen bonds between the water hydrogen atoms with the surface oxygen atoms, as described above. Differently from the molecular adsorption, the dissociative one is characterized by two hydroxyl stretching modes in the high frequency region without any bending mode signal. Thus, the experiments distinguish the molecular from the dissociative adsorption by the presence of the bending band. In the dissociated configurations, the two types of stretching modes correspond to the O_w_–H and O_2c_–H stretch. In the case of the intrapair dissociative adsorption, the O_w_–H and the O_2c_–H stretching frequencies are quite similar and greater than the symmetric and asymmetric frequencies of the molecular adsorption. This is equally valid when comparing the deuterated forms. Instead, in the case of the interpair dissociative adsorption, the O_2c_–H stretching frequency is comparable with the molecular adsorption, while the O_w_–H stretching frequency is much greater. This observation suggests a significant difference between the two types of dissociative interactions, which we think is due to the presence of a weak hydrogen bond between the O_2c_–H group and the oxygen atom in the O_w_–H adsorbate in the case of the interpair dissociative adsorption, as noted above from the distances values (Table 2). Same considerations can be applied to the deuterated case.

**Table 3:** Comparison of Harmonic Frequencies for H_2_O and D_2_O Adsorption on the TiO_2_ Anatase(101) Surface against Literature Data^a^

	layers	DFT *V*_*xc*_	θ	H_2_O			D_2_O		
Gas Phase									
Q-E present				1580	3731	3843	1156	2689	2815
Molecular Adsorption									
Q-E present	4L	PBE	0.25	1586	3580	3647	1163	2581	2673
Q-E^78^		PBE+U		1573	3702	3807	1153	2669	2791
Q-E^27^		PBE		1579	3611	3686			
DMol3^28^		PBE			3516	3620			
Intrapair Dissociative Adsorption									
					(O_w_–H)	(O_2c_–H)		(O_w_–D)	(O_2c_–D)
bond distance (Å)					0.976	0.973		0.976	0.973
Q-E present	4L	PBE	0.25		3760	3797		2738	2765
Q-E^78^		PBE+U			3750	3807		2726	2766
DMol3^28^		PBE			3645	3689			
VASP^23^		PBE+U				3734/3722			
Interpair Dissociative Adsorption									
					(O_w_–H)	(O_2c_–H)		(O_w_–D)	(O_2c_–D)
bond distance (Å)					0.975	0.982		0.975	0.982
Q-E present	4L	PBE	0.25		3775	3644		2748	2654

a All the frequencies are in cm^–1^.

### Classical versus Semiclassical Power Spectra

The harmonic approximation may not be accurate enough to predict experimental frequencies. In particular, the zero-point energy of the OH (OD) stretching mode is significant, so that the atomic motion is sensitive to the PES much above the minimum, in sharp contrast to the harmonic picture. We now employ the methods described above, that is, the quasi-classical and the semiclassical molecular dynamics ones, to gain a complete spectroscopic picture of the water molecule adsorption on the (101) anatase surface.

As a first improvement, we include classical anharmonic effects by calculating the quasi-classical power spectra with the time-averaging filtering technique of the velocity autocorrelation function, as described in eq 2. As a further improvement, the quantum mechanical power spectra are calculated using the DC-SCIVR method of eq 7. We recall that with the semiclassical method, we account for not only the classical and quantum anharmonicities, but also the combination and overtone vibrational states.

**Figure 2:**
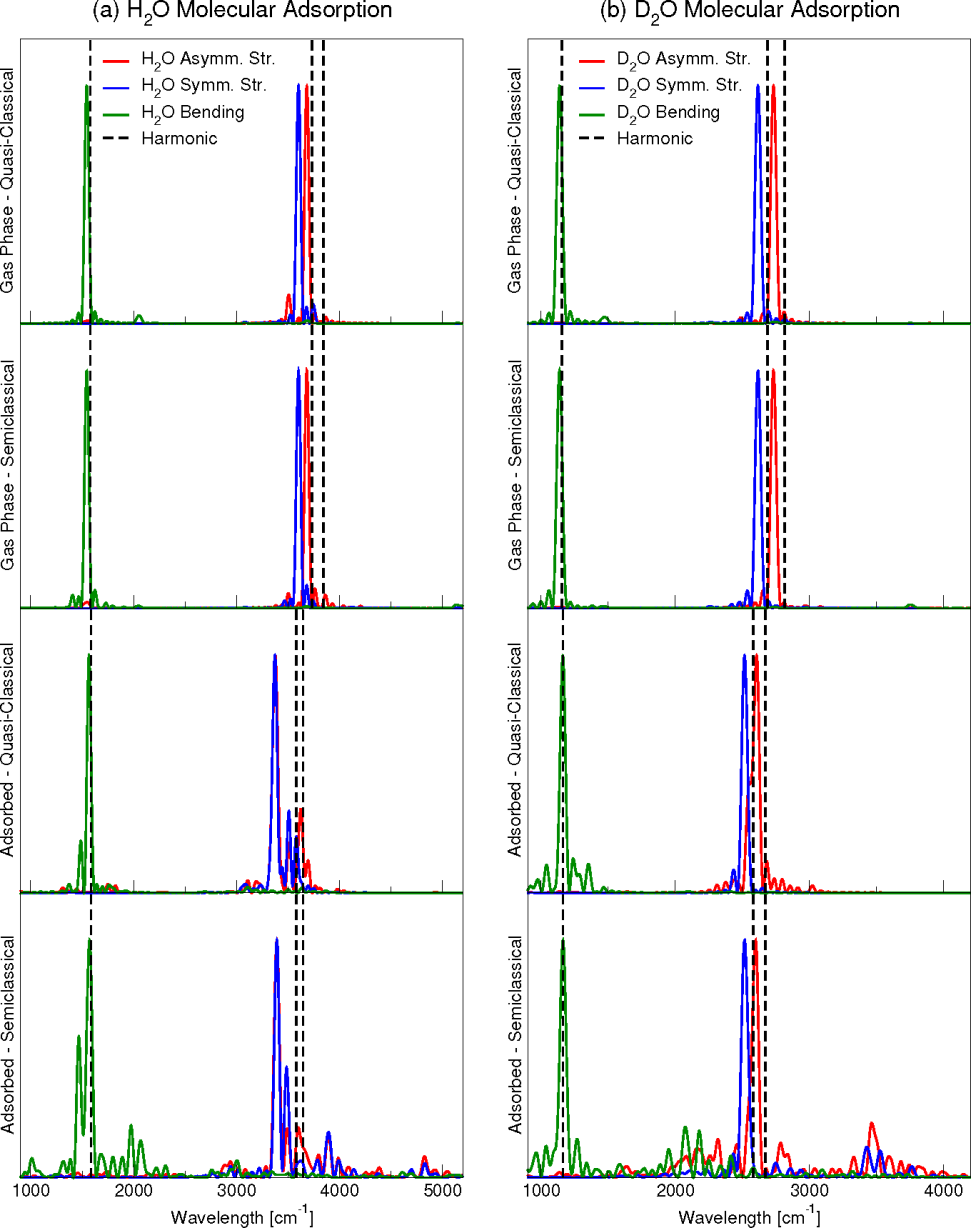
Comparison between quasi-classical and semiclassical spectra for H_2_O in gas phase and adsorbed on anatase(101). Left panel: H_2_O adsorption. Right panel: D_2_O adsorption. DC-SCIVR semiclassical calculations refer to a 14-dimensional subspace in the case of H_2_O and 12-dimensional subspace for D_2_O.

Figure 2 shows the molecular adsorption power spectra, both in the gas phase (before adsorption) and after adsorption. Frequencies are red-shifted after the inclusion of anharmonic effects in a greater amount for the hydrogenated system respect to the deuterated one, as expected. In the gas phase, the fundamental frequencies are equally reproduced by both methods. Instead, when moving to the adsorbed water molecule there is a significant reduction of the frequency splitting between the symmetric and asymmetric modes which is larger for H_2_O than D_2_O. In addition, the semiclassical power spectra of the molecular adsorbed water show numerous combination bands with the low-frequency phonons of the titania substrate. This information provided by DC-SCIVR clearly proves the coupling between molecular modes and phonons. Specifically, DC-SCIVR indicates that lower frequency phonons are more strongly coupled to adsorbed H_2_O than D_2_O.

Table 4 compares the results of Figure 2 with the available experimental results. Our Q-E computational setup is not so accurate for the gas phase as expected, since the PBE functional yields longer bonds and lower frequencies with respect to the experiments^83^ To check if this discrepancy is due to the plane-wave basis set, we perform a Gaussian basis sets harmonic, classical, and DC-SCIVR frequency calculation, and we find that this is consistent with our Q-E set up when adopting a PBE *V*_*xc*_. However, frequencies are slightly more accurate with a hybrid PBE0 *V*_*xc*_ and almost exact with a classical and DC SCIVR B3LYP *V*_*xc*_ calculation (see Tables S6 and S7 in the Supporting Information) when compared with the gas-phase experimental values. This shows that the discrepancy is mainly due to the PBE functional and not to the DC SCIVR method. As far as the adsorbate frequencies are concerned, our quasi-classical and semiclassical fundamentals are anyway consistent with the experimental literature (see Table 4), apart from the bending case. Nevertheless, upon adsorption, the amount of downshift from the harmonic estimate to the semiclassical values ranges from 200 to 250 cm^–1^, thus much larger than for the isolated molecule (130 cm^–1^). This shows that the anharmonic contributions are much larger upon adsorption than in the gas phase. We believe that this is a genuine characteristic that is largely independent of the actual functional that is employed, as we observed the same amount of anharmonic contributions for different functionals in the gas phase (see Tables S6 and S7 in the Supporting Information). In the deuterated system, the gas-phase frequencies are about 50 cm^–1^ off the experimental values, while no terms of experimental comparison are available for the adsorbed system. In this case, the anharmonic correction for the stretching is ∼70 cm^–1^ both in gas and condensed phases.

**Table 4:** Comparison of Vibrational Frequencies of H_2_O in the Gas Phase and after Adsorption on the TiO_2_ Anatase(101) Surface^a^

	molecular H_2_O			molecular D_2_O		
	bending	symmetric stretching	asymmetric stretching	bending	symmetric stretching	asymmetric stretching
	Gas Phase			Gas Phase		
experiment^79−82^	1595	3657	3756	1178	2671	2788
harmonic	1580	3731	3843	1156	2689	2815
quasi-classical	1545	3602	3683	1138	2617	2733
DC SCIVR	1546	3602	3682	1138	2618	2732
	Adsorbed			Adsorbed		
experiment	1615,^21^ 1635^19,20^	from 3300 to 3700^19,20^		1205–1210^18,21^		
harmonic	1586	3580	3647	1163	2581	2673
quasi-classical	1567	3373	3376	1162	2517	2607
DC SCIVR	1572	3392	3392	1164	2518	2600

a All the frequencies are in cm^–1^.

**Figure 3:**
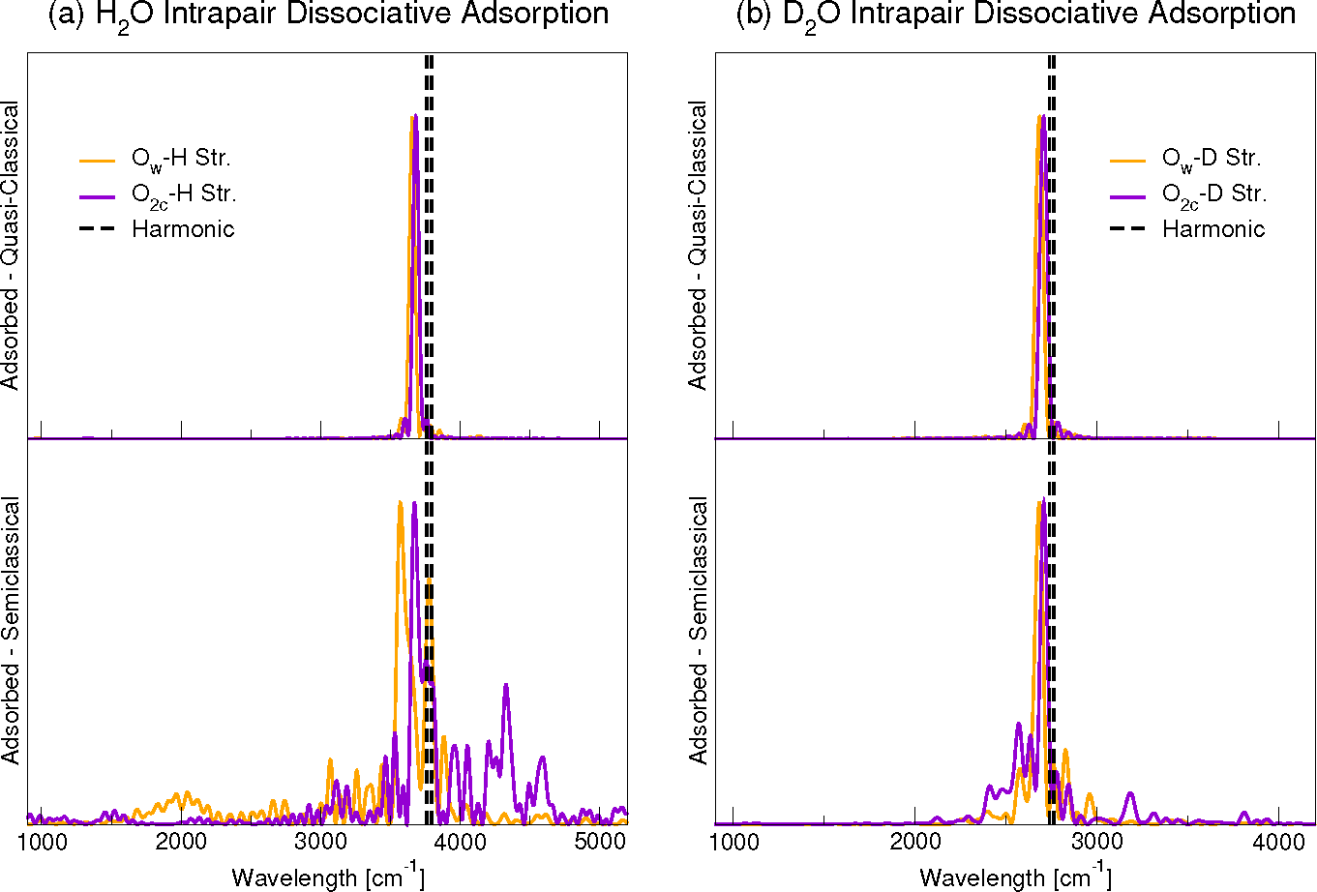
Comparison of quasi-classical and semiclassical spectra for H_2_O dissociative adsorption on anatase(101) surface in the intrapair configuration. Left: H_2_O adsorption; right: D_2_O adsorption. Semiclassical calculations refer to a 6-dimensional subspace for H_2_O and 5-dimensional subspace for D_2_O.

**Figure 4:**
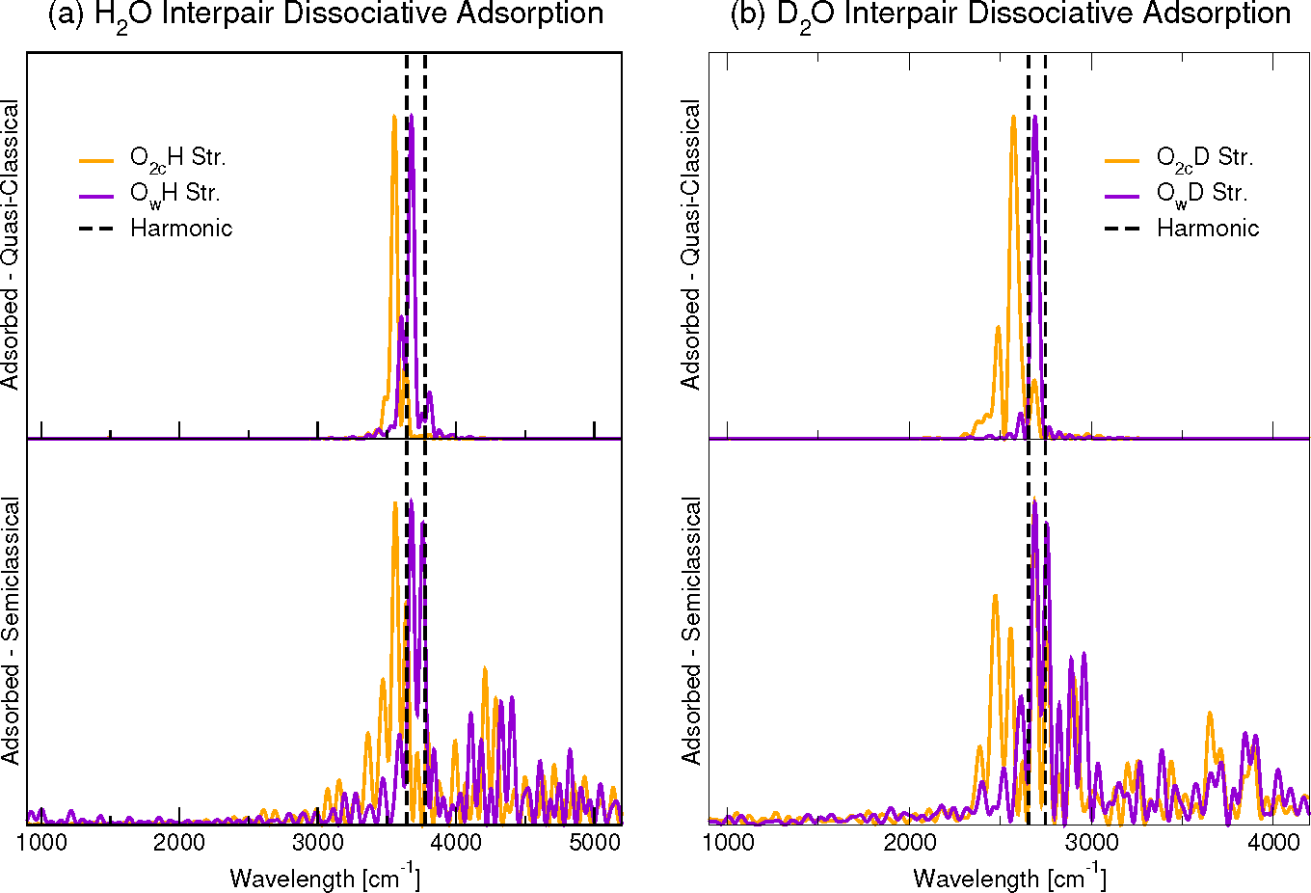
Same as in Figure 3 but for the interpair configuration. Semiclassical calculations refer to a 22-dimensional subspace for H_2_O and 14-dimensional subspace for D_2_O.

Figures 3 and 4 report the classical and semiclassical power spectra of the intra- and interpair adsorbates, respectively. In the case of the intrapair adsorption (Figure 3), the two O_w_–H and O_2c_–H stretching modes are characterized by almost equal frequencies, both for the hydrogenated and deuterated systems at the classical level of theory. The semiclassical simulation (bottom panels of Figure 3) shows a slightly lower frequency for the O_w_–H stretch than for to the O_2c_–H one, while such splitting is tiny for the deuterated case (Table 5). In addition, the semiclassical simulation yields a significant coupling between the water stretches and the phonon modes which is larger for the protonated than the deuterated surface. In particular, the O_2c_–H stretch is coupled to higher frequency phonons than the O_w_–H one.

**Table 5:** Comparison of Vibrational Frequencies of H_2_O Adsorption on the TiO_2_ Anatase(101) Surface in the Dissociative Geometry^a^

	dissociated H_2_O		dissociated D_2_O	
	O_w_–H stretching	O_2c_–H stretching	O_w_–D stretching	O_2c_–D stretching
	Intrapair Dissociative Adsorption			
bond distance (Å)	0.976	0.973	0.976	0.973
harmonic	3760	3797	2738	2765
quasi-classical	3657	3683	2684	2707
DC-SCIVR	3572	3674	2682	2708
	Interpair Dissociative Adsorption			
bond distance (Å)	0.975	0.982	0.975	0.982
harmonic	3775	3644	2748	2654
quasi-classical	3678	3555	2691	2573
DC-SCIVR (6-dim.)	3680	3550		
DC-SCIVR (14-dim.)			2690	2474–2558–2688
DC-SCIVR (22-dim.)	3678	3560		

a All the frequencies are in cm^–1^. The two DC SCIVR calculations for H_2_O in the interpair dissociative adsorption are performed respectively with a 6-dimensional (upper line) and a 22-dimensional (lower line) vibrational subspace for comparison.

**Figure 5:**
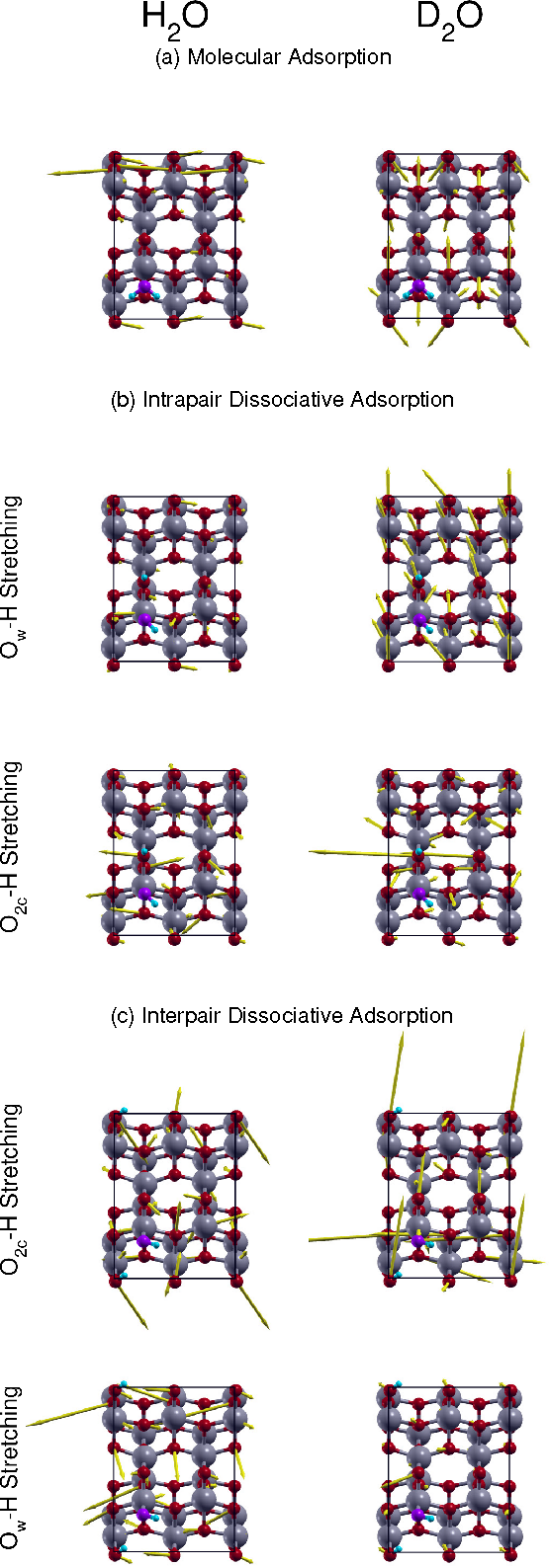
Displacement arrow plot for harmonic eigendisplacements of the phonon modes, which are coupled to the adsorbant modes, as obtained by inspection of the vibrational subspace grouping. The mass-scaled atomic displacement is larger the longer the arrow is of the corresponding atom.

Figure 4 shows the analogous spectra of Figure 3 but for the interpair dissociative adsorption. In this case, both at quasi-classical and semiclassical levels, we find a significant difference in frequency between the O_w_–H and the O_2c_–H stretching frequency. Also, by comparing the semiclassical results on the bottom panels of Figures 3 and 4, we can observe a significant involvement of the phonon modes in the interpair case respect to the intrapair one (as will be discussed in Adsorbate–Surface Interactions and shown in Figure 5). Specifically, beside the numerous combination peaks, the original streching peaks split both in classical and semiclassical simulations, suggesting the occurrence of Fermi-like resonances^84^ between the adsorbate and the titania.

A more detailed view on the frequency values is reported in Table 5 for the intra- and interpair dissociative adsorption of the hydrogenated and deuterated water molecule. No experimental values can be reported in this cases because the frequencies of the dissociative forms can not be assigned unequivocally, at variance with the molecular case, where the bending mode proves the presence of the ad-molecule.

As in the harmonic case, the main difference between the intra- and the interpair adsorption is that the fundamental O_w_–H and O_2c_–H stretching frequencies are very similar in the intrapair case, while in the interpair configuration the O_2c_–H one is lower in frequency. This is due to the H-bonding interaction. The amount of anharmonicity, which is comparable for classical and semiclassical calculations, is quite consistent and of the order of hundred of wavenumbers. In Table 5 we also consider the effect of the DC-SCIVR vibrational subspace dimensionality in the case of the interpair dissociative adsorption and show that, for a 6-dimensional subspace, results are almost the same as taking an almost 4× larger (22-dimensional) vibrational subspace.

In the case of the dissociative deuterated water adsorption, the computed frequencies are reported in Table 5. As anticipated from Figures 3 and 4, in this case the D–O stretching modes are strongly coupled with substrate phonons. In Table 5 we report the related frequency values. In this case the anharmonicity amount is about 50 cm^–1^ as in the case of the harmonic estimates, that is, reduced by the larger mass of the deuterion. In the case of the DC-SCIVR interpair calculations, we also checked that the choice of the subspace dimension does not affect our results. This consideration confirms the resonant interaction between D–O modes and the surface phonons.

### Adsorbate–Surface Interactions

An additional advantage of our DC-SCIVR methodology is the possibility of investigating the mechanism underlying the surface–adsorbate interactions by inspecting the vibrational subspace groups and in this way the coupling of surface phonons with the adsorbate normal modes. During the DC-SCIVR vibrational mode subspace determination, different normal mode groups are obtained for different averaged Hessian *H̅*_*ij*_ course-graining thresholds. By starting from high enough threshold values, where all subspaces are monodimensional, and then gradually decreasing the threshold value, we can detect which phonon modes share in primis the same subspace with the adsorbant modes. Figure 5 reports these modes for each adsorption scenario. The phonon modes are represented with a displacement arrow plot corresponding to the harmonic eigendisplacements. The mass-scaled atomic displacement is larger the longer is the arrow of the corresponding atom. Other examples are reported in the Supporting Information (Figures S3–S7). The atoms without any arrow either correspond to the adsorbated molecule or have not been relaxed during the optimization and are not part of the dynamics. These last atoms belong to the layers deeper than the second one.

All the phonon modes of Figure 5 are of the type of frustrated rotations respect to the adsorbant. In other words, these modes involve an almost rigid motion of the molecule with respect to the surface with an additional vibration of the surface atoms. On the top panel of Figure 5, we report the phonon mode that is specific to the water molecular adsorption process. It involves the surface atoms to which the hydrogen are bonded, the four O_2c_ surface sites, and for a smaller amount, the Ti_5c_ atoms to which the surface O_2c_ atoms are coordinated. We note that more many surface atoms are involved in the D_2_O molecular adsorption.

Both for the intrapair and the interpair dissociative adsorption, the surface modes that are coupled to the O_w_–H stretching implicate the surface O_2c_ and Ti_5c_ atom. These surface phonons show a more pronounced delocalization for the dissociated configurations (in particular, for the intrapair one) than in the case of molecular adsorption.

### Discussion

The results presented above are motivating us to provide new physical insight into the complex adsorption process of water molecules on titania anatase nanoparticles, mainly based on the comparison between experiments on one side and the classical and DC-SCIVR results on the other. The main open issue in the water adsorption is the identification of the hydroxyl stretching signals to be distinguished from the adsorbed molecular water one. The stretching of hydroxyl groups are generally attributed to higher frequencies compared to the molecular one and within the range 3600–3800 cm^–1^.^15,16,19−23^ However, the interpretation of the origin of the different bands is still controversial. There are two main interpretations that are based on the different explanation of the shift in the stretching frequency, which can be caused either by the oxygen coordination or by the differences in the OH bond length.^15,17,23,32,85−87^ Earlier work tried to distinguish between the two explanations relying on the spectral difference originated by the different oxygen coordination. Specifically, lower energy bands were assigned to the vibration of bridged hydroxyl groups (which in our case corresponds to the O_2c_–H stretching frequencies), while higher frequency modes were assigned to terminal hydroxyls (in our case, O_w_–H stretchings). No criteria are provided for the molecular O–H stretching modes. However, alternative explanations correlate the aforementioned bands to the OH bond length, either adsorbed in the dissociated form or within the water ad-molecule. Actually, there are several factors that conspire to determine the OH bond length, among which the oxygen coordination number or the presence of hydrogen bonds (with other water ad-molecules or surface oxygen atoms).^26,88^

We start our analysis from the molecular adsorption that is experimentally detected through the presence of the bending peak in the range 1615–1640 cm^–1^.^15−23^ Our calculations provide for this type of adsorption a small underestimation of the bending frequency, which is evident already at the harmonic level for the isolated H_2_O molecule and in a smaller amount for D_2_O as well. The underestimation is typical of the PBE functional. Therefore, rather than discussing the values for the frequencies for the adsorbed species, we will focus on the shift (usually negative) existing between the frequencies that are obtained in the quasi-classical or semiclassical levels with respect to the harmonic calculations. Such a shift is indeed a reliable quantity that is largely independent of the employed functional and the algorithms for solving the Kohn–Sham equations (see Supporting Information, Tables S6 and S7). Actually, it is essential to take into account the anharmonic contributions to the experimental spectra since the zero-point energy contribution (*hν*_s_/2) of the OH stretching mode in the water molecule corresponds to more than 2000 K of kinetic energy. This implies that the vibrational motion is sensitive to the high part of the potential energy surface (rather than its minimum), which is highly anharmonic. Experimental spectra exhibit several spectroscopy features within the 2500–3800 cm^–1^ range, where signals can be attributed not only to water stretches, but also to hydroxyl stretching modes that originate from water dissociation or from the synthesis procedure. As a confirmation, in the case of the molecular adsorption where we can identify the bending signal, the stretching frequencies have been assigned in the range 2500–3600 cm^–1^,^15−23^ making it impossible to identify the hydroxyl stretching signal or exclude its presence. In the stretching case, our classical and semiclassical results are consistent with the experimental range, while the harmonic estimates fall outside the upper frequency limit.

Eventually, we obtain the following comprehensive picture of the water adsorption process. The symmetric and the asymmetric stretches of the molecular adsorption and the O_2c_–H in the interpair dissociative adsorption configurations share similar frequencies because the H atoms are hydrogen-bonded to the nearby oxygen atoms. These frequencies are red-shifted with respect to the O_2c_–H of the intrapair and the O_w_–H of both the intra- and interpair adsorptions. Noteworthy, both classical and semicalssical dynamics results show degenerate frequencies for the adsorbed molecular symmetric and asymmetric stretching. This is due to the lower molecular symmetry after adsorption and to the strong coupling of the OH stretching to the surface modes, mainly frustrated rotations and translations, as obtained from the analysis of the vibrational eigenfunctions. In the intrapair dissociative configuration, the O_2c_–H stretching frequencies (both at the quasi- and semiclassical levels) are slightly higher with respect to their counterparts for the other adsorption configurations, which we attribute to the lack of hydrogen bonding. Generally, same considerations apply to D_2_O adsorption with the main difference between the deuterated water adsorption process and the hydrogenated one is unveiled by the DC-SCIVR spectra, where the Fermi resonances with the phonon bath is more evident in the deuterated case and in the interpair adsorption than in the other geometries, consistently with a strong coupling with the surface phonons for this case. Additionally, the eigenmode displacements suggest that in the molecular case the interactions are more local than in the dissociative one.

Finally, the previous analysis applies to the isolated water molecules. In the case of higher water coverage, we guess that more complex interactions, including hydrogen bonding between adsorbed molecules, or between ad-molecules and surface oxygen ions, would likely enhance anharmonic effects, making it even harder to disentangle all the individual contributions to the experimental spectra.

## Conclusions

In this work we aim at gaining further insights into water adsorption on the TiO_2_ anatase(101) surface. The systems is extremely complex. First, the nanoparticle morphology is far from the stoichiometric surface period slab model usually adopted in theoretical simulations. Second, nanoparticles are usually composed by two titania polymorphs, that is, rutile and anatase. Third, nanoparticles expose different type of surfaces and most of the time these surfaces are defective ones. Clearly, defects can play an important role in the kinetic and thermodynamics of water adsorption. Similarly, the presence of nanoparticles edges and corners can modify the interaction with the adsorbate. Fourth, one should include the intramolecular interaction between several adsorbed molecules which can be significant in case of high coverage. All these considerations do not allow to straightforwardly compare experimental measurements with simulations, where usually the adsorption process is described not considering all the possible experimental scenarios and more often limited to a single-molecule adsorption. However, if one sticks on the experimental evidence, it is not possible to reach a definitive conclusion about the water atomic configuration for the adsorption on anatase(101) surface. Experimentalists tried to assign the water adsorption structure by comparing IR spectra of nanoparticles with different surface ratios^21^ or by inspecting IR spectra outgassed at different temperatures.^15,17,23^

For all these reasons, we decided to tackle this system with a novel computational approach, which is based on quantum anharmonic vibrational calculations. Eventually, we think that we have been able to provide clear spectroscopic information that can allow experimentalists to devise new experimental setups and disentagle the different types of water adsorption. We believe that the information hereby provided will be very helpful to determine which is the most stable water adsorption configuration if new experimental conditions are devised, such as ultravacuum ones.

## Supporting Information Available

Functional dependency of the geometrical and vibrational properties in gas phase and upon adsorption, convergence of vibrational frequencies against the number of k-points and slab thickness, figures of adsorption geometries with periodic boundary conditions, and eigendisplacement plots for additional coupled modes (PDF)

## References

[ref1] Chen H.; Nanayakkara C. E.; Grassian V. H. Titanium Dioxide Photocatalysis in Atmospheric Chemistry. Chem. Rev. 2012, 112, 5919–5948. 10.1021/cr3002092. 23088691

[ref2] Kudo A.; Miseki Y. Heterogeneous photocatalyst materials for water splitting. Chem. Soc. Rev. 2009, 38, 253–278. 10.1039/B800489G. 19088977

[ref3] Osterloh F. E. Inorganic nanostructures for photoelectrochemical and photocatalytic water splitting. Chem. Soc. Rev. 2013, 42, 2294–2320. 10.1039/C2CS35266D. 23072874

[ref4] Diebold U. The surface science of titanium dioxide. Surf. Sci. Rep. 2003, 48, 53–229. 10.1016/S0167-5729(02)00100-0.

[ref5] Selloni A. In Handbook of Materials Modeling: Applications: Current and Emerging Materials; Andreoni W., Yip S., Eds.; Springer International Publishing, 2018; pp 1–23.

[ref6] Luo C.; Ren X.; Dai Z.; Zhang Y.; Qi X.; Pan C. Present Perspectives of Advanced Characterization Techniques in TiO_2_-Based Photocatalysts. ACS Appl. Mater. Interfaces 2017, 9, 23265–23286. 10.1021/acsami.7b00496. 28628307

[ref7] Sun C.; Liu L.-M.; Selloni A.; Lu G. Q. M.; Smith S. C. Titania-water interactions: a review of theoretical studies. J. Mater. Chem. 2010, 20, 10319–10334. 10.1039/c0jm01491e.

[ref8] De Angelis F.; Di Valentin C.; Fantacci S.; Vittadini A.; Selloni A. Theoretical Studies on Anatase and Less Common TiO_2_ Phases: Bulk, Surfaces, and Nanomaterials. Chem. Rev. 2014, 114, 9708–9753. 10.1021/cr500055q. 24926899

[ref9] Guo Q.; Zhou C.; Ma Z.; Ren Z.; Fan H.; Yang X. Elementary photocatalytic chemistry on TiO_2_ surfaces. Chem. Soc. Rev. 2016, 45, 3701–3730. 10.1039/C5CS00448A. 26335268

[ref10] Bourikas K.; Kordulis C.; Lycourghiotis A. Titanium Dioxide (Anatase and Rutile): Surface Chemistry, Liquid-Solid Interface Chemistry, and Scientific Synthesis of Supported Catalysts. Chem. Rev. 2014, 114, 9754–9823. 10.1021/cr300230q. 25253646

[ref11] Lo Presti L.; Pifferi V.; Di Liberto G.; Cappelletti G.; Falciola L.; Cerrato G.; Ceotto M. Direct measurement and modeling of spontaneous charge migration across anatase–brookite nanoheterojunctions. J. Mater. Chem. A 2021, 9, 7782–7790. 10.1039/D1TA01040A.

[ref12] Mu R.; Zhao Z.-J.; Dohnálek Z.; Gong J. Structural motifs of water on metal oxide surfaces. Chem. Soc. Rev. 2017, 46, 1785–1806. 10.1039/C6CS00864J. 28180223

[ref13] Björneholm O.; Hansen M. H.; Hodgson A.; Liu L.-M.; Limmer D. T.; Michaelides A.; Pedevilla P.; Rossmeisl J.; Shen H.; Tocci G.; et al. Water at Interfaces. Chem. Rev. 2016, 116, 7698–7726. 10.1021/acs.chemrev.6b00045. 27232062

[ref14] Wang Y.; Wöll C. IR spectroscopic investigations of chemical and photochemical reactions on metal oxides: bridging the materials gap. Chem. Soc. Rev. 2017, 46, 1875–1932. 10.1039/C6CS00914J. 28221385

[ref15] Finnie K. S.; Cassidy D. J.; Bartlett J. R.; Woolfrey J. L. IR Spectroscopy of Surface Water and Hydroxyl Species on Nanocrystalline TiO_2_ Films. Langmuir 2001, 17, 816–820. 10.1021/la0009240.

[ref16] Maira A.; Coronado J.; Augugliaro V.; Yeung K.; Conesa J.; Soria J. Fourier Transform Infrared Study of the Performance of Nanostructured TiO_2_ Particles for the Photocatalytic Oxidation of Gaseous Toluene. J. Catal. 2001, 202, 413–420. 10.1006/jcat.2001.3301.

[ref17] Soria J.; Sanz J.; Sobrados I.; Coronado J. M.; Maira A. J.; Hernandez-Alonso M. D.; Fresno F. FTIR and NMR Study of the Adsorbed Water on Nanocrystalline Anatase. J. Phys. Chem. C 2007, 111, 10590–10596. 10.1021/jp071440g.

[ref18] Belhadj H.; Hakki A.; Robertson P. K. J.; Bahnemann D. W. In situ ATR-FTIR study of H_2_O and D_2_O adsorption on TiO_2_ under UV irradiation. Phys. Chem. Chem. Phys. 2015, 17, 22940–22946. 10.1039/C5CP03947A. 26266701

[ref19] Sheng H.; Zhang H.; Song W.; Ji H.; Ma W.; Chen C.; Zhao J. Activation of Water in Titanium Dioxide Photocatalysis by Formation of Surface Hydrogen Bonds: An In Situ IR Spectroscopy Study. Angew. Chem., Int. Ed. 2015, 54, 5905–5909. 10.1002/anie.201412035. 25809908

[ref20] Mino L.; Pellegrino F.; Rades S.; Radnik J.; Hodoroaba V.-D.; Spoto G.; Maurino V.; Martra G. Beyond Shape Engineering of TiO_2_ Nanoparticles: Post-Synthesis Treatment Dependence of Surface Hydration, Hydroxylation, Lewis Acidity and Photocatalytic Activity of TiO_2_ Anatase Nanoparticles with Dominant 001 or 101 Facets. ACS Appl. Nano Mater. 2018, 1, 5355–5365. 10.1021/acsanm.8b01477.

[ref21] Zhang H.; Zhou P.; Chen Z.; Song W.; Ji H.; Ma W.; Chen C.; Zhao J. Hydrogen-Bond Bridged Water Oxidation on 001 Surfaces of Anatase TiO2. J. Phys. Chem. C 2017, 121, 2251–2257. 10.1021/acs.jpcc.6b11900.

[ref22] Litke A.; Su Y.; Tranca I.; Weber T.; Hensen E. J. M.; Hofmann J. P. Role of Adsorbed Water on Charge Carrier Dynamics in Photoexcited TiO_2_. J. Phys. Chem. C 2017, 121, 7514–7524. 10.1021/acs.jpcc.7b00472. PMC5388900 28413570

[ref23] Mahdavi-Shakib A.; Arce-Ramos J. M.; Austin R. N.; Schwartz T. J.; Grabow L. C.; Frederick B. G. Frequencies and Thermal Stability of Isolated Surface Hydroxyls on Pyrogenic TiO2 Nanoparticles. J. Phys. Chem. C 2019, 123, 24533–24548. 10.1021/acs.jpcc.9b05699.

[ref24] Mino L.; Morales-García A.; Bromley S. T.; Illas F. Understanding the nature and location of hydroxyl groups on hydrated titania nanoparticles. Nanoscale 2021, 13, 6577–6585. 10.1039/D1NR00610J. 33885537

[ref25] Chi M.; Sun X.; Lozano-Blanco G.; Tatarchuk B. J. XPS and FTIR investigations of the transient photocatalytic decomposition of surface carbon contaminants from anatase TiO2 in UHV starved water/oxygen environments. Appl. Surf. Sci. 2021, 570, 151147 10.1016/j.apsusc.2021.151147.

[ref26] Haque F.; Finocchi F.; Chenot S.; Jupille J.; Stankic S. Interplay between Single and Cooperative H_2_ Adsorption in the Saturation of Defect Sites at MgO Nanocubes. J. Phys. Chem. C 2018, 122, 17738–17747. 10.1021/acs.jpcc.8b03192.

[ref27] Gala F.; Agosta L.; Zollo G. Water Kinetics and Clustering on the (101) TiO_2_ Anatase Surface. J. Phys. Chem. C 2016, 120, 450–456. 10.1021/acs.jpcc.5b10934.

[ref28] Posternak M.; Baldereschi A.; Delley B. Dissociation of Water on Anatase TiO_2_ Nanoparticles: the Role of Undercoordinated Ti Atoms at Edges. J. Phys. Chem. C 2009, 113, 15862–15867. 10.1021/jp9032113.

[ref29] Manzhos S.; Ihara M. Computational vibrational spectroscopy of molecule-surface interactions: what is still difficult and what can be done about it. Phys. Chem. Chem. Phys. 2022, na 10.1039/d2cp01389d. 35543373

[ref30] Jug K.; Nair N. N.; Bredow T. Molecular dynamics investigation of water adsorption on rutile surfaces. Surf. Sci. 2005, 590, 9–20. 10.1016/j.susc.2005.05.055.

[ref31] Kavathekar R. S.; Dev P.; English N. J.; MacElroy J. Molecular dynamics study of water in contact with the TiO2 rutile-110, 100, 101, 001 and anatase-101, 001 surface. Mol. Phys. 2011, 109, 1649–1656. 10.1080/00268976.2011.582051.

[ref32] Arrouvel C.; Digne M.; Breysse M.; Toulhoat H.; Raybaud P. Effects of morphology on surface hydroxyl concentration: a DFT comparison of anatase-TiO_2_ and gamma-alumina catalytic supports. J. Catal. 2004, 222, 152–166. 10.1016/j.jcat.2003.10.016.

[ref33] Benoit D. M. Vibrational Signature of a Single Water Molecule Adsorbed on Pt(111): Toward a Reliable Anharmonic Description. J. Phys. Chem. A 2015, 119, 11583–11590. 10.1021/acs.jpca.5b08543. 26535801

[ref34] Ceotto M.; Di Liberto G.; Conte R. Semiclassical ”Divide-and-Conquer” Method for Spectroscopic Calculations of High Dimensional Molecular Systems. Phys. Rev. Lett. 2017, 119, 010401 10.1103/PhysRevLett.119.010401. 28731742

[ref35] Di Liberto G.; Conte R.; Ceotto M. Divide and conquer” semiclassical molecular dynamics: A practical method for spectroscopic calculations of high dimensional molecular systems. J. Chem. Phys. 2018, 148, 014307 10.1063/1.5010388. 29306274

[ref36] Cazzaniga M.; Micciarelli M.; Moriggi F.; Mahmoud A.; Gabas F.; Ceotto M. Anharmonic calculations of vibrational spectra for molecular adsorbates: A divide-and-conquer semiclassical molecular dynamics approach. J. Chem. Phys. 2020, 152, 104104 10.1063/1.5142682. 32171221

[ref37] Giannozzi P.; Baroni S.; Bonini N.; Calandra M.; Car R.; Cavazzoni C.; Ceresoli D.; Chiarotti G. L.; Cococcioni M.; Dabo I.; et al. QUANTUM ESPRESSO: a modular and open-source software project for quantum simulations of materials. J. Phys.-Conddens. Mater. 2009, 21, 395502 10.1088/0953-8984/21/39/395502. 21832390

[ref38] Giannozzi P.; Andreussi O.; Brumme T.; Bunau O.; Nardelli M. B.; Calandra M.; Car R.; Cavazzoni C.; Ceresoli D.; Cococcioni M.; et al. Advanced capabilities for materials modelling with Quantum ESPRESSO. J. Phys.: Condens. Mater. 2017, 29, 465901 10.1088/1361-648X/aa8f79. 29064822

[ref39] Baroni S.; de Gironcoli S.; Dal Corso A.; Giannozzi P. Phonons and related crystal properties from density-functional perturbation theory. Rev. Mod. Phys. 2001, 73, 515–562. 10.1103/RevModPhys.73.515.

[ref40] Rognoni A.; Conte R.; Ceotto M. Caldeira-Leggett model vs ab initio potential: A vibrational spectroscopy test of water solvation. J. Chem. Phys. 2021, 154, 094106 10.1063/5.0040494. 33685187

[ref41] Di Liberto G.; Conte R.; Ceotto M. Divide-and-conquer” semiclassical molecular dynamics: An application to water clusters. J. Chem. Phys. 2018, 148, 104302 10.1063/1.5023155. 29544263

[ref42] Micciarelli M.; Conte R.; Suarez J.; Ceotto M. Anharmonic vibrational eigenfunctions and infrared spectra from semiclassical molecular dynamics. J. Chem. Phys. 2018, 149, 064115 10.1063/1.5041911. 30111132

[ref43] Micciarelli M.; Gabas F.; Conte R.; Ceotto M. An Effective Semiclassical Approach to IR Spectroscopy. J. Chem. Phys. 2019, 150, 184113 10.1063/1.5096968. 31091908

[ref44] Gabas F.; Di Liberto G.; Conte R.; Ceotto M. Protonated glycine supramolecular systems: the need for quantum dynamics. Chem. Sci. 2018, 9, 7894–7901. 10.1039/C8SC03041C. 30542548 PMC6237109

[ref45] Gabas F.; Di Liberto G.; Ceotto M. Vibrational investigation of nucleobases by means of divide and conquer semiclassical dynamics. J. Chem. Phys. 2019, 150, 224107 10.1063/1.5100503. 31202241

[ref46] Conte R.; Parma L.; Aieta C.; Rognoni A.; Ceotto M. Improved semiclassical dynamics through adiabatic switching trajectory sampling. J. Chem. Phys. 2019, 151, 214107 10.1063/1.5133144. 31822104

[ref47] Aieta C.; Micciarelli M.; Bertaina G.; Ceotto M. Anharmonic quantum nuclear densities from full dimensional vibrational eigenfunctions with application to protonated glycine. Nat. Commun. 2020, 11, 1–9. 10.1038/s41467-020-18211-3. 32859910 PMC7455743

[ref48] Bertaina G.; Di Liberto G.; Ceotto M. Reduced rovibrational coupling Cartesian dynamics for semiclassical calculations: Application to the spectrum of the Zundel cation. J. Chem. Phys. 2019, 151, 114307 10.1063/1.5114616. 31542046

[ref49] Aieta C.; Bertaina G.; Micciarelli M.; Ceotto M. Representing molecular ground and excited vibrational eigenstates with nuclear densities obtained from semiclassical initial value representation molecular dynamics. J. Chem. Phys. 2020, 153, 214117 10.1063/5.0031391. 33291909

[ref50] Botti G.; Ceotto M.; Conte R. On-the-fly adiabatically switched semiclassical initial value representation molecular dynamics for vibrational spectroscopy of biomolecules. J. Chem. Phys. 2021, 155, 234102 10.1063/5.0075220. 34937370

[ref51] Gabas F.; Conte R.; Ceotto M. Quantum Vibrational Spectroscopy of Explicitly Solvated Thymidine in Semiclassical Approximation. J. Phys. Chem. Lett. 2022, 13, 1350–1355. 10.1021/acs.jpclett.1c04087. 35109652 PMC8842300

[ref52] Conte R.; Ceotto M. Quantum Chemistry and Dynamics of Excited States: Methods and Applications; John Wiley & Sons, Ltd., 2020; p 595.

[ref53] Ceotto M.; Atahan S.; Shim S.; Tantardini G. F.; Aspuru-Guzik A. First-principles semiclassical initial value representation molecular dynamics. Phys. Chem. Chem. Phys. 2009, 11, 3861–3867. 10.1039/b820785b. 19440613

[ref54] Ceotto M.; Atahan S.; Tantardini G. F.; Aspuru-Guzik A. Multiple coherent states for first-principles semiclassical initial value representation molecular dynamics. J. Chem. Phys. 2009, 130, 234113 10.1063/1.3155062. 19548717

[ref55] Ceotto M.; Dell’Angelo D.; Tantardini G. F. Multiple coherent states semiclassical initial value representation spectra calculations of lateral interactions for CO on Cu (100). J. Chem. Phys. 2010, 133, 054701 10.1063/1.3462242. 20707543

[ref56] Ceotto M.; Tantardini G. F.; Aspuru-Guzik A. Fighting the curse of dimensionality in first-principles semiclassical calculations: Non-local reference states for large number of dimensions. J. Chem. Phys. 2011, 135, 214108 10.1063/1.3664731. 22149780

[ref57] Ceotto M.; Valleau S.; Tantardini G. F.; Aspuru-Guzik A. First principles semiclassical calculations of vibrational eigenfunctions. J. Chem. Phys. 2011, 134, 234103 10.1063/1.3599469. 21837839

[ref58] Conte R.; Aspuru-Guzik A.; Ceotto M. Reproducing Deep Tunneling Splittings, Resonances, and Quantum Frequencies in Vibrational Spectra From a Handful of Direct Ab Initio Semiclassical Trajectories. J. Phys. Chem. Lett. 2013, 4, 3407–3412. 10.1021/jz401603f. 26705583

[ref59] Heller E. J. The semiclassical way to molecular spectroscopy. Acc. Chem. Res. 1981, 14, 368–375. 10.1021/ar00072a002.

[ref60] Heller E. J. Frozen Gaussians: A very simple semiclassical approximation. J. Chem. Phys. 1981, 75, 2923–2931. 10.1063/1.442382.

[ref61] Heller E. J. Cellular dynamics: A new semiclassical approach to time-dependent quantum mechanics. J. Chem. Phys. 1991, 94, 2723–2729. 10.1063/1.459848.

[ref62] Shalashilin D. V.; Child M. S. Multidimensional quantum propagation with the help of coupled coherent states. J. Chem. Phys. 2001, 115, 5367–5375. 10.1063/1.1394939.

[ref63] Herman M. F.; Kluk E. A semiclassical justification for the use of non-spreading wavepackets in dynamics calculations. Chem. Phys. 1984, 91, 27–34. 10.1016/0301-0104(84)80039-7.

[ref64] Kluk E.; Herman M. F.; Davis H. L. Comparison of the propagation of semiclassical frozen Gaussian wave functions with quantum propagation for a highly excited anharmonic oscillator. J. Chem. Phys. 1986, 84, 326–334. 10.1063/1.450142.

[ref65] Di Liberto G.; Conte R.; Ceotto M. Divide and conquer” semiclassical molecular dynamics: A practical method for spectroscopic calculations of high dimensional molecular systems. J. Chem. Phys. 2018, 148, 014307 10.1063/1.5010388. 29306274

[ref66] Gandolfi M.; Rognoni A.; Aieta C.; Conte R.; Ceotto M. Machine learning for vibrational spectroscopy via divide-and-conquer semiclassical initial value representation molecular dynamics with application to N-methylacetamide. J. Chem. Phys. 2020, 153, 204104 10.1063/5.0031892. 33261493

[ref67] Zhao Z.; Li Z.; Zou Z. Understanding the interaction of water with anatase TiO_2_ (101) surface from density functional theory calculations. Phys. Lett. A 2011, 375, 2939–2945. 10.1016/j.physleta.2011.06.022.

[ref68] Zhao Z.; Li Z.; Zou Z. A. Theoretical Study of Water Adsorption and Decomposition on the Low-Index Stoichiometric Anatase TiO_2_ Surfaces. J. Phys. Chem. C 2012, 116, 7430–7441. 10.1021/jp212407s.

[ref69] Aschauer U.; He Y.; Cheng H.; Li S.-C.; Diebold U.; Selloni A. Influence of Subsurface Defects on the Surface Reactivity of TiO_2_: Water on Anatase (101). J. Phys. Chem. C 2010, 114, 1278–1284. 10.1021/jp910492b.

[ref70] Aschauer U. J.; Tilocca A.; Selloni A. Ab initio simulations of the structure of thin water layers on defective anatase TiO_2_(101) surfaces. Int. J. Quantum Chem. 2015, 115, 1250–1257. 10.1002/qua.24918.

[ref71] He Y.; Tilocca A.; Dulub O.; Selloni A.; Diebold U. Local ordering and electronic signatures of submonolayer water on anatase TiO_2_(101). Nat. Mater. 2009, 8, 585 10.1038/nmat2466. 19465917

[ref72] Martinez-Casado R.; Mallia G.; Harrison N. M.; Pérez R. First-Principles Study of the Water Adsorption on Anatase(101) as a Function of the Coverage. J. Phys. Chem. C 2018, 122, 20736–20744. 10.1021/acs.jpcc.8b05081.

[ref73] Agosta L.; Gala F.; Zollo G. Water diffusion on TiO_2_ anatase surface. AIP Conf. Proc. 2015, 1667, 020006 10.1063/1.4922562.

[ref74] Vittadini A.; Selloni A.; Rotzinger F.; Grätzel M. Structure and energetics of water adsorbed at TiO2 anatase (101) and (001) surfaces. Phys. Rev. Lett. 1998, 81, 2954 10.1103/PhysRevLett.81.2954.

[ref75] Petersen T.; Klüner T. Water Adsorption on Ideal Anatase-TiO2(101): An Embedded Cluster Model for Accurate Adsorption Energetics and Excited State Properties. Z. Phys. Chem. 2020, 234, 813–834. 10.1515/zpch-2019-1425.

[ref76] Selli D.; Fazio G.; Seifert G.; Di Valentin C. Water Multilayers on TiO2 (101) Anatase Surface: Assessment of a DFTB-Based Method. J. Chem. Theory Comput. 2017, 13, 3862–3873. 10.1021/acs.jctc.7b00479. 28679048 PMC5562391

[ref77] Egashira M.; Kawasumi S.; Kagawa S.; Seiyama T. Temperature Programmed Desorption Study of Water Adsorbed on Metal Oxides. I. Anatase and Rutile. B. Chem. Soc. Jpn. 1978, 51, 3144–3149. 10.1246/bcsj.51.3144.

[ref78] Dette C.; Pérez-Osorio M. A.; Mangel S.; Giustino F.; Jung S. J.; Kern K. Single-Molecule Vibrational Spectroscopy of H_2_O on Anatase TiO_2_(101). J. Phys. Chem. C 2017, 121, 1182–1187. 10.1021/acs.jpcc.6b10379.

[ref79] NIST Computational Chemistry Comparison and Benchmark Database, NIST Standard Reference Database Number 101, Release 19, April, 2018, Johnson R. D. III, Ed.; http://cccbdb.nist.gov/.

[ref80] Shimanouchi T. Tables of Molecular Vibrational Frequencies Consolidated; National Bureau of Standard, 1972; Vol. I.

[ref81] Huber G. K. P.; Herzberg Molecular Spectra and Molecular Structure IV. Constants of Diatomic Molecules; Springer: New York, NY, 1979.

[ref82] Varetti E. A far infrared and theoretical ab initio vibrational study of fluorosulfonic acid as monomer and cyclic dimer. J. Mol. Struc.-THEOCHEM 1998, 429, 121–130. 10.1016/S0166-1280(97)00347-3.

[ref83] Xu X.; Goddard W. A. Bonding Properties of the Water Dimer: A Comparative Study of Density Functional Theories. J. Phys. Chem. A 2004, 108, 2305–2313. 10.1021/jp035869t.

[ref84] Gygi F. m. c. Ab initio molecular dynamics in adaptive coordinates. Phys. Rev. B 1995, 51, 11190–11193. 10.1103/PhysRevB.51.11190. 9977839

[ref85] Liu Q.; Liu L.; Xiao W. Doping Effects on the Adsorption of a Nitric Oxide Molecule on an Anatase (101) Surface. ChemPhysChem 2017, 18, 653–661. 10.1002/cphc.201601164. 28070929

[ref86] Chizallet C.; Digne M.; Arrouvel C.; Raybaud P.; Delbecq F.; Costentin G.; Che M.; Sautet P.; Toulhoat H. Insights into the Geometry, Stability and Vibrational Properties of OH Groups on c-Al2O3, TiO 2-Anatase and MgO from DFT Calculations. Top. Catal. 2009, 52, 1005–1016. 10.1007/s11244-009-9262-9.

[ref87] Dzwigaj S.; Arrouvel C.; Breysse M.; Geantet C.; Inoue S.; Toulhoat H.; Raybaud P. DFT makes the morphologies of anatase-TiO_2_ nanoparticles visible to IR spectroscopy. J. Catal. 2005, 236, 245–250. 10.1016/j.jcat.2005.09.034.

[ref88] Finocchi F.; Haque F.; Chenot S.; Jupille J.; Stankic S. Water dissociation on the low-coordinated sites of MgO nanopowders. J. Mater. Res. 2019, 34, 408–415. 10.1557/jmr.2018.461.

