# Supplementary material for: Quantum Anharmonic Calculations of Vibrational Spectra for Water Adsorbed on Titania Anatase(101) Surface: Dissociative versus Molecular Adsorption
Source: J Phys Chem C Nanomater Interfaces. 2022 Jul 19;126(29):12060–73. doi: 10.1021/acs.jpcc.2c02137 (PMC9340806; doi:10.1021/acs.jpcc.2c02137)
Supplement: Supplementary file 1 — jp2c02137_si_001.pdf [file jp2c02137_si_001.pdf]

Supplementary Information for: Quantum Anharmonic Calculations of Vibrational Spectra for Water Adsorbed on Titania Anatase (101) Surface: Dissociative versus Molecular Adsorption

Marco Cazzaniga,[*,†] Marco Micciarelli,[†] Fabio Gabas,[†] Fabio Finocchi,[‡] and Michele Ceotto[*,†]

†*Dipartimento di Chimica, Università degli Studi di Milano, via Golgi 19, 20133 Milano, Italy.*

‡*Sorbonne Université, CNRS, Institut des NanoSciences de Paris (INSP), 4 Place Jussieu, Paris F- 75005, France.*

E-mail: marco.cazzaniga@unimi.it; michele.ceotto@unimi.it

Table S1: Effects of the U parameter and the dispersion terms on the relevant geometrical coordinates and binding energies for molecular H_2O adsorption on the TiO_2 anatase (101) surface. The value chosen for the Hubbard U is 3.3 eV.

	Layers	DFT V_{xc}		r(Ti_{5c}–O_w) [Å]	r(O_w–H) [Å]	d(O_{2c}-H) [Å]	α(H-O_w-H) [°]	BE[a] [eV]
Q-E present	4L	PBE	θ=0.25	2.289	0.984	2.284	103.89	0.76
Q-E present	4L	PBE+U	θ=0.25	2.289	0.986	2.240/2.237	103.54	0.84
Q-E present	4L	PBE-D3	θ=0.25	2.284	0.986	2.184	103.45	1.00

[a]Experimental value 0.5-0.7 eV.[1]

Table S2: Effects of the U parameter and the dispersion terms on the relevant geometrical coordinates and binding energies for dissociative H_2O adsorption on the TiO_2 anatase (101) surface. The value chosen for the Hubbard U is 3.3 eV.

	Layers	DFT V_{xc}	θ	r(Ti_{5c}–O_w) [Å]	r(O_w–H) [Å]	d(O_{2c}-H) [Å]	d(O_w-H_{diss}) [Å]	α(O_w-H_{diss}-O_{2c}) [°]	BE [eV]
				**Intrapair Dissociative Adsorption**					
Q-E present	4L	PBE	0.25	1.824	0.976	0.973	2.680	79.578	0.41
Q-E present	4L	PBE+U	0.25	1.848	0.977	0.972	2.685	80.048	0.55
Q-E present	4L	PBE-D3	0.25	1.822	0.977	0.972	2.735	78.015	0.57
				**Interpair Dissociative Adsorption**					
Q-E present	4L	PBE	0.25	1.854	0.975	0.982	2.487	138.282	0.29
Q-E present	4L	PBE+U	0.25	1.885	0.976	0.983	2.410	139.841	0.43
Q-E present	4L	PBE-D3	0.25	1.863	0.976	0.984	2.331	142.441	0.51

Table S3: Comparison of harmonic frequencies for H_2O and D_2O adsorbed on the TiO_2 anatase (101) surface for different computational set-up. Finite Difference calculations have been performed with an atomic displacements of 0.01 Å and the frequencies are in cm^{-1}.

	Layers	DFT	θ	Method		**H_2O**			**D_2O**	
		V_{xc}			**Molecular Adsorption**					
Q-E present	4L	PBE	0.25	DFPT	1586	3580	3647	1163	2581	2673
Q-E present	4L	PBE	0.25	FD	1578	3580	3670	1158	2583	2685
Q-E present	4L	PBE+U	0.25	FD	1582	3549	3635	1161	2562	2659
Q-E present	4L	PBE-D3	0.25	FD	1587	3560	3621	1164	2565	2653
					**Intrapair Dissociative Adsorption**					
						(O_w-H)	(O_{2c}-H)		($O_w - D$)	($O_{2c} - D$)
Q-E present	4L	PBE	0.25	DFPT		3760	3797		2738	2765
Q-E present	4L	PBE	0.25	FD		3745	3760		2731	2738
Q-E present	4L	PBE+U	0.25	FD		3752	3753		2731	2737
Q-E present	4L	PBE-D3	0.25	FD		3748	3753		2731	2734
					**Interpair Dissociative Adsorption**					
						(O_w-H)	(O_{2c}-H)		($O_w - D$)	($O_{2c} - D$)
Q-E present	4L	PBE	0.25	DFPT		3775	3644		2748	2654
Q-E present	4L	PBE	0.25	FD		3736	3614		2723	2634
Q-E present	4L	PBE+U	0.25	FD		3718	3581		2710	2610
Q-E present	4L	PBE-D3	0.25	FD		3717	3554		2709	2591

Table S4: Convergence test of the harmonic frequencies *vs* the k-point sampling of the Brillouin zone. All the frequencies are in cm^{-1}.

	**Molecular H_2O**			**Intrapair H_2O**		**Interpair H_2O**	
Γ-point only	1586	3580	3647	3760	3797	3775	3644
2×2×1	1589	3582	3647	3746	3793	3784	3663
4×4×1	1589	3581	3647	3746	3793	3784	3663

Table S5: Convergence test of the harmonic frequencies *vs* the slab thickness. All the frequencies are in cm^{-1}.

	**Molecular H_2O**			**Intrapair H_2O**		**Interpair H_2O**	
4 Layers	1586	3580	3647	3760	3797	3775	3644
6 Layers	1586	3579	3645	3761	3795	3772	3653
8 Layers	1588	3577	3643	3761	3795	3769	3650

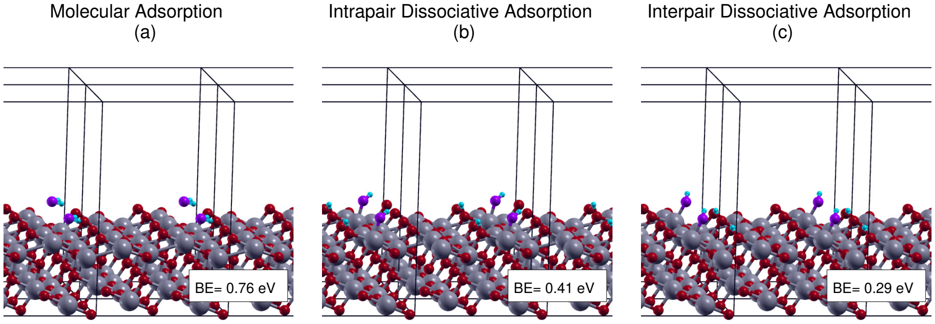

Figure S1: Supercell optimized geometries of molecular and dissociative H_2O adsorbtion on the TiO_2 anatase (101) surface. Titanium atoms are in gray, surface oxygen in red, molecular oxygen in violet and hydrogen in cyan.

Table S6: Comparison of vibrational frequencies of H_2O in gas phase. All the frequencies are in cm^{-1}.

Gas Phase Molecular H_2O							
		Bending		Symmetric Stretching		Asymmetric Stretching	
		Frequency	Δ	Frequency	Δ	Frequency	Δ
Experiment[2–4]		1595		3657		3756	
Q-E PBE	Harmonic	1580		3731		3843	
	Quasi-classical	1545	-35	3602	-129	3683	-160
	DC SCIVR	1546	-34	3602	-129	3682	-161
NWChem PBE	Harmonic	1605		3707		3809	
	Quasi-classical	1577	-28	3587	-120	3657	-152
	DC SCIVR	1578	-27	3588	-119	3658	-151
NWChem PBE0	Harmonic	1641		3874		3975	
	Quasi-classical	1612	-29	3758	-116	3831	-144
	DC SCIVR	1612	-29	3758	-116	3832	-143
NWChem B3LYP	Harmonic	1638		3807		3905	
	Quasi-classical	1609	-29	3691	-116	3756	-149
	DC SCIVR	1608	-30	3690	-117	3756	-149

Table S7: Comparison of vibrational frequencies of D_2O in gas phase. All the frequencies are in cm^{-1}.

**Gas Phase Molecular** D_2O							
		Bending		Symmetric Stretching		Asymmetric Stretching	
		Frequency	Δ	Frequency	Δ	Frequency	Δ
Experiment [2,3,5]		1178		2671		2788	
Q-E PBE	Harmonic	1156		2689		2815	
	Quasi-classical	1138	-18	2617	-72	2733	-82
	DC SCIVR	1138	-18	2618	-71	2732	-83
NWChem PBE	Harmonic	1174		2673		2789	
	Quasi-classical	1159	-15	2607	-66	2713	-76
	DC SCIVR	1160	-14	2608	-65	2712	-77
NWChem PBE0	Harmonic	1200		2793		2912	
	Quasi-classical	1185	-15	2730	-63	2839	-73
	DC SCIVR	1186	-14	2730	-63	2838	-74
NWChem B3LYP	Harmonic	1198		2745		2860	
	Quasi-classical	1183	-15	2680	-65	2786	-74
	DC SCIVR	1182	-16	2680	-65	2786	-74

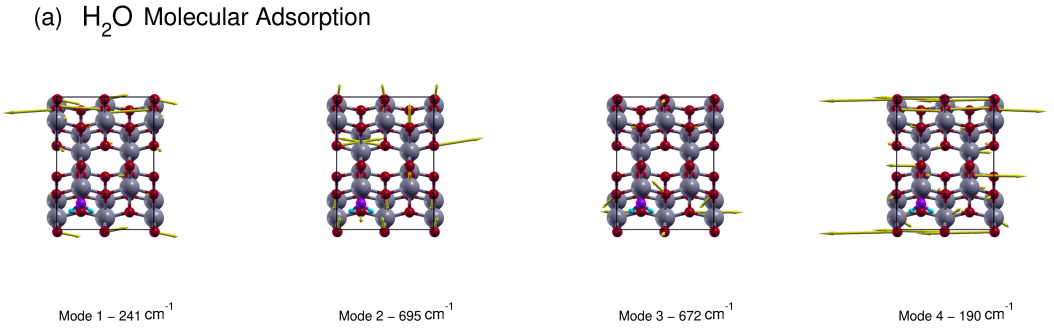

Figure S2: Plot of normalized eigen-displacements for the first 4 modes entering in the subspace for molecular adsorbed H_2O.

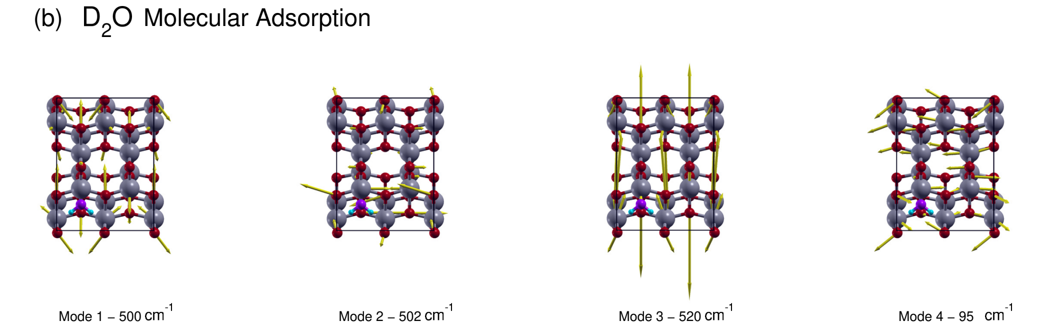

Figure S3: Plot of normalized eigen-displacements for the first 4 modes entering in the subspace for molecular adsorbed D_2O.

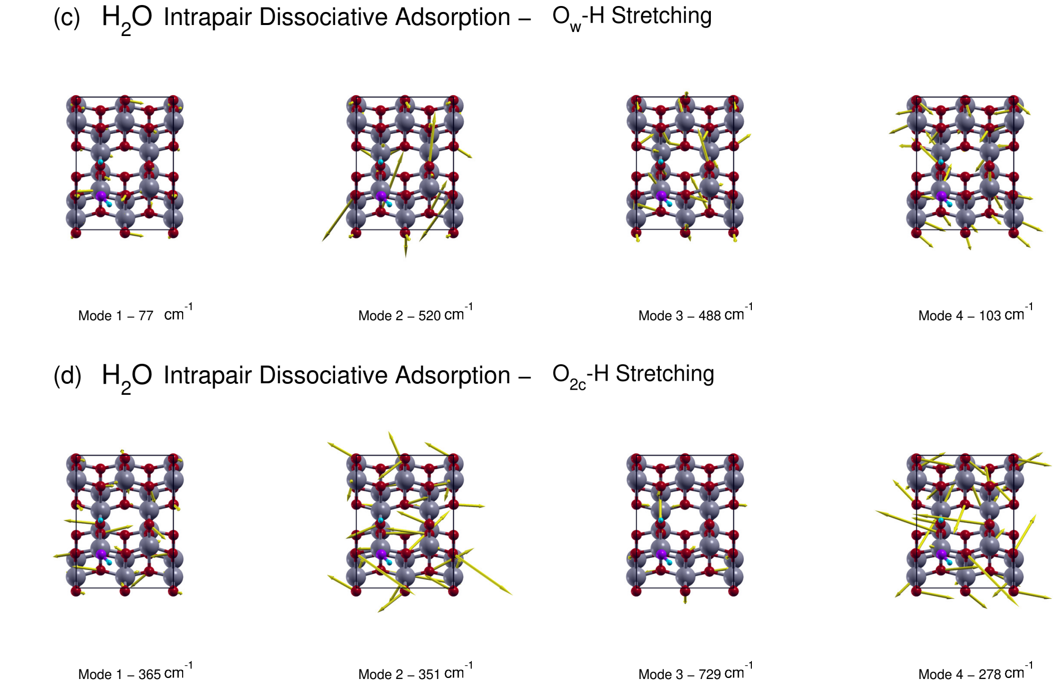

Figure S4: Plot of normalized eigen-displacements for the first 4 modes entering in the subspace for intrapair dissociative adsorbed H_2O.

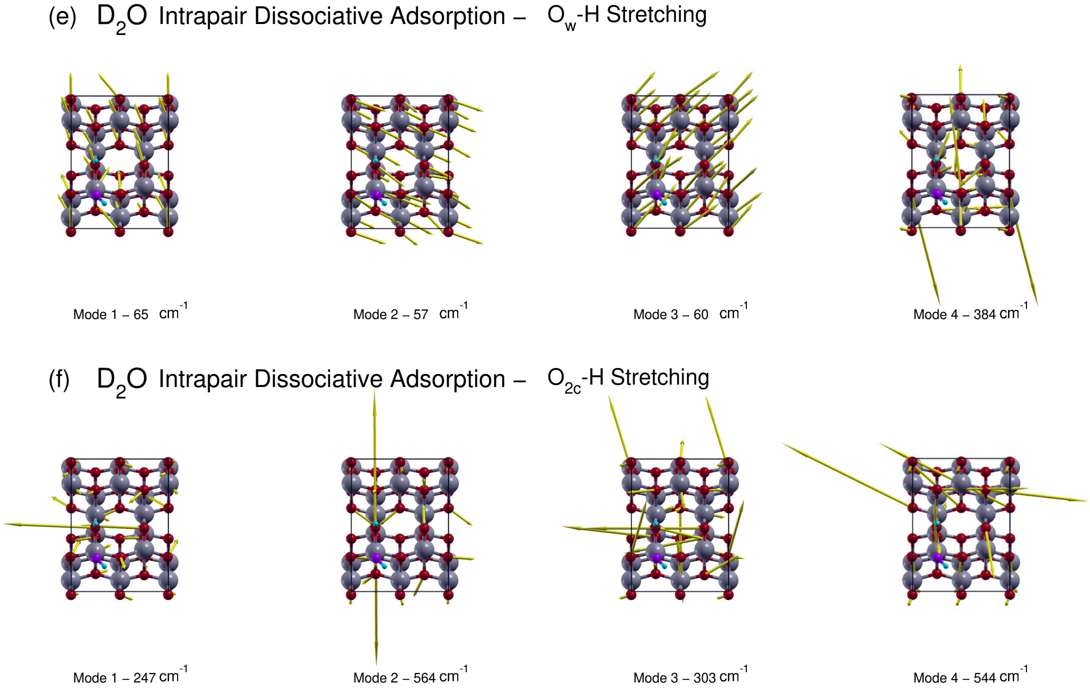

Figure S5: Plot of normalized eigen-displacements for the first 4 modes entering in the subspace for intrapair dissociative adsorbed D_2O.

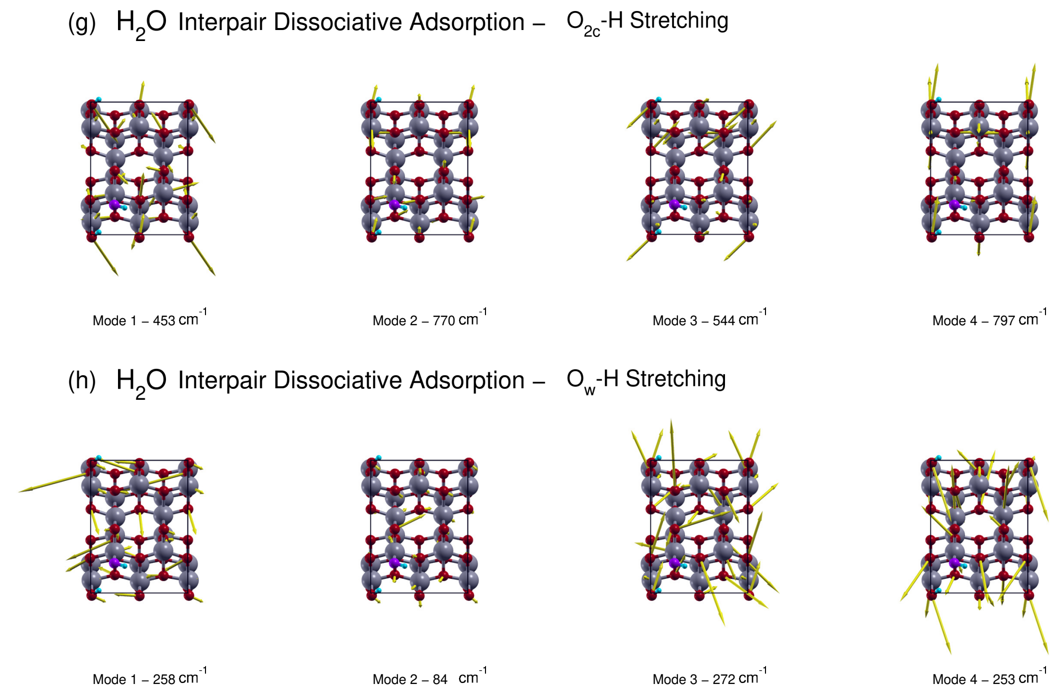

Figure S6: Plot of normalized eigen-displacements for the first 4 modes entering in the subspace for interpair dissociative adsorbed H_2O.

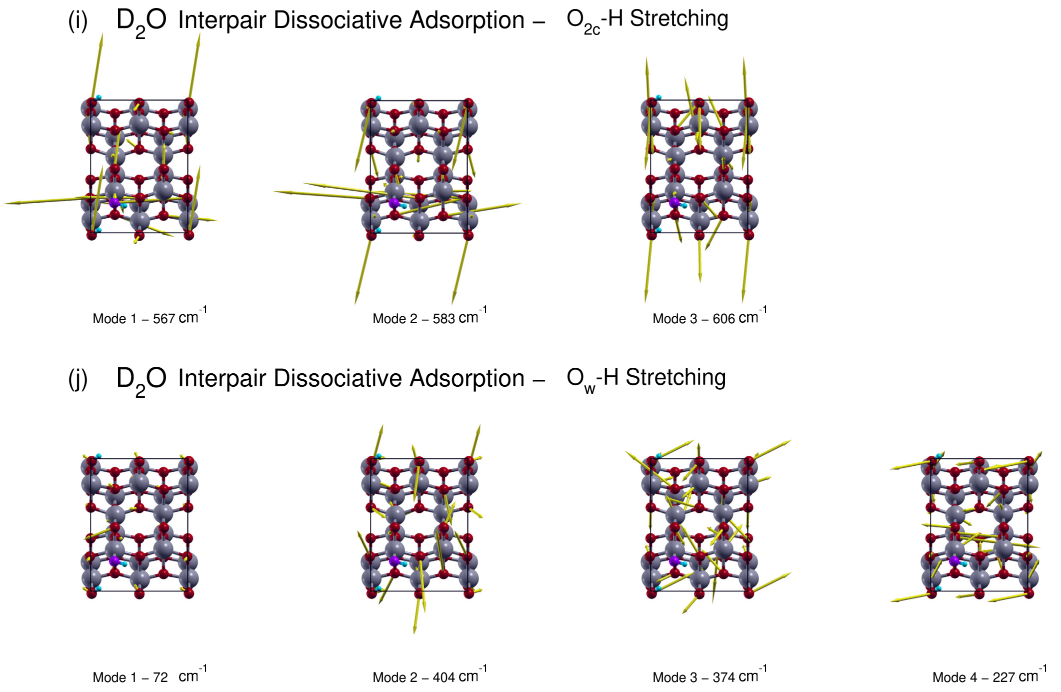

Figure S7: Plot of normalized eigen-displacements for the first 4 modes entering in the subspace for the interpair dissociative adsorbed D_2O.

References

(1) Egashira, M.; Kawasumi, S.; Kagawa, S.; Seiyama, T. Temperature Programmed Desorption Study of Water Adsorbed on Metal Oxides. I. Anatase and Rutile. *B. Chem. Soc. Jpn.* **1978**, *51*, 3144–3149.

(2) NIST Computational Chemistry Comparison and Benchmark Database, NIST Standard Reference Database Number 101, Release 19, April 2018, Editor: Russell D. Johnson III, http://cccbdb.nist.gov/.

(3) Shimanouchi, T. *Tables of Molecular Vibrational Frequencies Consolidated, Volume I*; National Bureau of Standard, 1972.

(4) Huber, G., K. P.; Herzberg *Molecular Spectra and Molecular Structure IV. Constants of Diatomic Molecules*; Springer New York, NY, 1979.

(5) Varetti, E. A far infrared and theoretical ab initio vibrational study of fluorosulfonic acid as monomer and cyclic dimer. *J. Mol. Struc.-THEOCHEM* **1998**, *429*, 121–130.
